# Optimizing Management to Reduce the Mortality of COVID-19: Experience From a Designated Hospital for Severely and Critically Ill Patients in China

**DOI:** 10.3389/fmed.2021.582764

**Published:** 2021-03-10

**Authors:** Wei Zhu, Huaqiu Zhang, Yong Li, Zeyang Ding, Zhuo Liu, Yajun Ruan, Huan Feng, Ganxun Li, Bo Liu, Fan He, Ning Zhou, Jiangang Jiang, Zhixiang Wen, Gang Xu, Jianping Zhao, Bixiang Zhang, Daowen Wang, Zhouping Tang, Hui Wang, Jihong Liu

**Affiliations:** ^1^Department of Emergency and Critical Care Medicine, Tongji Hospital, Tongji Medical College, Huazhong University of Science and Technology, Wuhan, China; ^2^Department of Neurosurgery, Tongji Hospital, Tongji Medical College, Huazhong University of Science and Technology, Wuhan, China; ^3^Department of Respiratory and Critical Care Medicine, Tongji Medical College, Huazhong University of Science and Technology, Wuhan, China; ^4^Hepatic Surgery Center and Hubei Key Laboratory of Hepato-Biliary-Pancreatic Diseases, Tongji Hospital, Tongji Medical College, Huazhong University of Science and Technology, Wuhan, China; ^5^Department of Urology, Tongji Hospital, Tongji Medical College, Huazhong University of Science and Technology, Wuhan, China; ^6^Department of Oncology, Tongji Hospital, Tongji Medical College, Huazhong University of Science and Technology, Wuhan, China; ^7^Department of Nephrology, Department of Internal Medicine, Tongji Hospital, Tongji Medical College, Huazhong University of Science and Technology, Wuhan, China; ^8^Department of Cardiology, Department of Internal Medicine and Genetic Diagnosis Center, Tongji Hospital, Tongji Medical College, Huazhong University of Science and Technology, Wuhan, China; ^9^Institute of Organ Transplantation, Tongji Hospital, Tongji Medical College, Huazhong University of Science and Technology, Wuhan, China; ^10^Department of Neurology, Tongji Hospital, Tongji Medical College, Huazhong University of Science and Technology, Wuhan, China; ^11^Department of Obstetrics and Gynecology, Tongji Hospital, Tongji Medical College, Huazhong University of Science and Technology, Wuhan, China

**Keywords:** COVID-19, management, experience, mortality, severely and critically ill

## Abstract

**Background:** The coronavirus disease 2019 (COVID-19) has swept through the world at a tremendous speed, and there is still limited data available on the treatment for COVID-19. The mortality of severely and critically ill COVID-19 patients in the Optical Valley Branch of Tongji Hospital was low. We aimed to analyze the available treatment strategies to reduce mortality.

**Methods:** In this retrospective, single-center study, we included 1,106 COVID-19 patients admitted to the Optical Valley Branch of Tongji Hospital from February 9 to March 9, 2020. Cases were analyzed for demographic and clinical features, laboratory data, and treatment methods. Outcomes were followed up until March 29, 2020.

**Results:** Inflammation-related indices (hs-CRP, ESR, serum ferritin, and procalcitonin) were significantly higher in severe and critically ill patients than those in moderate patients. The levels of cytokines, including IL-6, IL2R, IL-8, and TNF-α, were also higher in the critical patients. Incidence of acute respiratory distress syndrome (ARDS) in the severely and critically ill group was 23.0% (99/431). Sixty-one patients underwent invasive mechanical ventilation. The correlation between SpO_2_/FiO_2_ and PaO_2_/FiO_2_ was confirmed, and the cut-off value of SpO_2_/FiO_2_ related to survival was 134.43. The mortality of patients with low SpO_2_/FiO_2_ (<134.43) at intubation was higher than that of patients with high SpO_2_/FiO_2_ (>134.43) (72.7 vs. 33.3%). Among critical patients, the application rates of glucocorticoid therapy, continuous renal replacement therapy (CRRT), and anticoagulation treatment reached 55.2% (238/431), 7.2% (31/431), and 37.1% (160/431), respectively. Among the intubated patients, the application rates of glucocorticoid therapy, CRRT, and anticoagulation treatment were respectively 77.0% (47/61), 54.1% (33/61), and 98.4% (60/61).

**Conclusion:** No vaccines or specific antiviral drugs for COVID-19 have been shown to be sufficiently safe and effective to date. Comprehensive treatment including ventilatory support, multiple organ function preservation, glucocorticoid use, renal replacement therapy, anticoagulation, and restrictive fluid management was the main treatment strategy. Early recognition and intervention, multidisciplinary collaboration, multi-organ function support, and personalized treatment might be the key for reducing mortality.

## Introduction

Coronavirus disease 2019 (COVID-19), caused by SARS-CoV-2, has spread at a tremendous rate around the world (1). The World Health Organization (WHO) has declared it a public health emergency of international concern. As of September 16, 2020, the number of patients infected with SARS-CoV-2 has exceeded 7,266,074 globally, and more than 935 792 have died, with case fatality rates reaching more than 10% in some countries (2–9). This rapidly spreading pandemic has become a serious threat to worldwide health.

Relatively few studies have described the clinical characteristics of COVID-19 patients in countries such as United States and Italy (7–9). Although most COVID-19 patients have asymptomatic or mild disease with a good prognosis, a few cases may rapidly develop severe disease with high risk of mortality, and have to receive treatment in intensive care units (ICU) (5, 7). According to a report on 44 672 cases from the Chinese Center for Disease Control and Prevention (CDC), the mortality of the critical cases was 49.0% (6). Especially in those who developed acute respiratory distress syndrome (ARDS), the mortality can reach as high as 52.4~64.7% (4–7). Therefore, reducing the mortality of severe COVID-19 patients is urgent and can save many lives.

During the outbreak of COVID-19 in China, the mild and moderate COVID-19 cases were transported and treated in Fangcang shelter hospitals, while the severe cases and critically ill patients were transferred to designated hospitals (10). Although the Optical Valley Branch of Tongji Hospital (Wuhan, China) was a designated hospital for severely and critically ill COVID-19 patient, the mortality of the severely and critically ill patients was 10.4%, and that of critically ill patients was 39.6%, which was lower than published data (4, 6, 11). To explore possible measures to reduce the mortality of severely and critically ill COVID-19 patients, in this study we retrospectively analyzed our therapeutic process, hoping to provide more evidence for better COVID-19 treatment.

## Methods

### Study Design and Participants

This single-center, retrospective, observational study was conducted at the Optical Valley Branch of Tongji Hospital of Tongji Medical College, Huazhong University of Science and Technology. During the outbreak of COVID-19, the Optical Valley Branch of Tongji Hospital was reconstructed and designated as a hospital for treatment of severely and critically ill COVID-19 patients by the Chinese government from February 9, 2020 to March 30, 2020. The diagnosis and classification of the severity of COVID-19 was conducted according to the guidance for COVID-19 (the 7th version) established by the National Health Commission of China (12). Laboratory-confirmed cases with admission dates from February 9, 2020 to March 9, 2020 were included in our study. Patients younger than 18 years old, or lacking core sets of medical data like blood tests, or for whom the entire hospital stay lasted for <12 h were excluded from further analysis. This study was carried out under the authorization of the National Health Commission of China, and it was approved by the Ethics Commission of Tongji hospital (Approval No.: TJ-IRB20200334), and the written informed consent requirement was waived for anonymized data in view of the rapidly emerging infectious disease.

### Data Collection and Definitions

We obtained epidemiological, demographic, clinical, laboratory, management, and outcome data from patients' medical records in the Tongji Cloud Hospital Information System (HIS) using standardized data collection forms. Clinical outcomes were followed up until March 29, 2020. The most intense level of oxygen support during hospitalization [nasal cannula, non-invasive mechanical ventilation (NMV), invasive mechanical ventilation (IMV), or IMV with extracorporeal membrane oxygenation (ECMO) were recorded]. Records of anticoagulant therapy, systemic glucocorticoid therapy, and continuous renal replacement therapy were also collected. All data were checked by two clinicians (ZD and HF). If there was any difference in recording and interpreting the data by the two primary reviewers, the third researcher (GL) checked and adjudicated the difference.

ARDS and sepsis were defined according to the interim guidance of the WHO (13), while acute kidney injury (AKI), cardiac injury, acute heart failure, and acute liver injury were defined as described previously (14).

### Laboratory and Radiological Measurements

The majority of the baseline clinical data was collected from the first day of admission. To diagnose COVID-19, respiratory specimens including pharyngeal swabs or sputum samples of patients were collected, and tested by real-time RT-PCR for SARS-CoV-2 as described previously, and/or blood tests for SARS-CoV-2-specific IgM and IgG antibodies. In addition, respiratory specimens were also tested to exclude the presence of other respiratory virus infections, including influenza virus A and B, respiratory syncytial virus, parainfluenza virus, and adenovirus. Initial laboratory tests included a complete blood count, coagulation profile, and serum biochemical tests [including liver and renal function, electrolytes, creatinine kinase, lactate dehydrogenase (LDH), and cytokines]. All patients had at least a chest X-ray or computed tomography (CT) scan on admission and/or during their hospital stay. The association of SpO_2_/FiO_2_ with mortality of intubated patients was analyzed, and in these patients, values of SpO_2_/FiO_2_ were measured 0.5h before intubation. PaO_2_/FiO_2_ were measured within 1 h before intubation. If repeated measurements of SpO_2_/FiO_2_ values were performed in this time period, mean levels were calculated and used for further analysis. Frequency of examinations was determined by clinicians treating COVID-19 patients.

### Statistical Analysis

All statistical analyses were performed using R software, version 3.6.2 (R Foundation for Statistical Computing, Vienna, Austria), or SPSS 21.0 (IBM Corp, Armonk, NY, USA). Categorical variables were expressed using frequencies and percentages. Continuous variables were shown using the median (IQR) or mean (SD) values. Means for continuous variables were compared using the independent-samples *t*-test when the data were normally distributed; if not, the Mann–Whitney test was used. Proportions for categorical variables were compared using chi-squared and Fisher's exact tests. Descriptive analyses was performed for demographic, clinical, and laboratory data. Cumulative rates of in-hospital mortality were determined using the Kaplan–Meier method. The cut-off value of SpO_2_/FiO_2_ was confirmed using a maximally selected log-rank statistic. The ability of SpO_2_/FiO_2_ to distinguish non-survivors from survivors was also assessed by estimating the area under the ROC curve (AUC) using the method developed by DeLong et al. (15). Based on the oxygen dissociation curve, a linear fitting model was constructed to evaluate the relationship between SpO_2_/FiO_2_ and the oxygenation index of patients. Differences with *P* < 0.05 were considered statistically significant.

### Role of the Funding Source

The corresponding authors had full access to all the data of the study and had final responsibility for the decision to submit for publication.

## Results

From February 9 to March 9, 2020, a total of 675 patients with moderate disease and 431 severely or critically ill COVID-19 patients were admitted to the Optical Valley Branch of Tongji Hospital (designated hospital for severely and critically ill COVID-19 patients). The overall mortality was 5.2% (58/1106), the mortality of severe and critically ill patients was 10.4% (45/431), and the mortality for critically ill patients was 39.6% (42/106) (Supplementary Table 6).

### Demographics and Baseline Characteristics of COVID-19 Patients

As shown in Table 1, the severely ill patients (median age 65 years, range 23–92) were older than the patients with moderate disease (61 years, range 18–95). Of the 675 patients with moderate disease, 304 were male (45.0%), and 371 were female (55.0%). Of the 431 severely and critically ill patients, 220 were male (51.0%) and 211 were female (49.0%). The percentage of patients with preexisting conditions, including hypertension, diabetes, and chronic pulmonary disease among the severely and critically ill patients was higher than in patients with moderate disease [145 (33.6%) vs. 191 (28.3%), 80 (18.6%) vs. 90 (13.3%), 39 (9.0%) vs. 40 (5.9%)].

**Table 1:** Baseline characteristics of COVID-2019 patients enrolled in this study.

	**All** **(*N* = 1,106)**	**Moderate** **(*n* = 675)**	**Severe +** **Critically ill**	***P*** **value**
			**(*n* = 431)**	
**Demographic characteristics**				
Age- yr	63 [18–95]	61 [18–95]	65 [23–92]	<0.001
Age≥ 65	482 (43.6)	264 (39.1)	218 (50.6)	<0.001
Gender-Female	582 (52.6)	371 (55.0)	211 (49.0)	0.056
Duration from illness onset to admission	15 [10, 22]	16 [10, 23]	15 [10, 21]	0.114
**Personal history**				
Smoking history	15 (1.4)	8 (1.2)	7 (1.6)	0.598
Current smoker	9 (0.8)	6 (0.9)	3 (0.7)	1
Former smoker	6 (0.5)	2 (0.3)	4 (0.9)	0.216
**Coexisting disorder**				
Cardiovascular disease	87 (7.9)	48 (7.1)	39 (9.0)	0.253
Hypertension	336 (30.4)	191 (28.3)	145 (33.6)	0.061
Diabetes	170 (15.4)	90 (13.3)	80 (18.6)	0.021
Cerebrovascular disease	34 (3.1)	17 (2.5)	17 (3.9)	0.212
Chronic pulmonary disease	79 (7.1)	40 (5.9)	39 (9.0)	0.055
Chronic kidney disease	40 (3.6)	27 (4.0)	13 (3.0)	0.415
Chronic liver disease	95 (8.6)	59 (8.7)	36 (8.4)	0.912
Malignancy	46 (4.2)	28 (4.1)	18 (4.2)	1
**Other respiratory pathogen infection- no. /total no. (%)**				
Other viruses	104 (9.4)	56 (8.3)	48 (11.1)	0.139
**Symptoms**				
Fever	784 (70.9)	460 (68.1)	324 (75.2)	0.012
Cough	862 (77.9)	516 (76.4)	346 (80.3)	0.138
Expectoration	641 (58.0)	380 (56.3)	261 (60.6)	0.17
Shortness of breath	472 (42.7)	266 (39.4)	206 (47.8)	0.006
Pharyngalgia	90 (8.1)	46 (6.8)	44 (10.2)	0.055
Rhinorrhoea	42 (3.8)	15 (2.2)	27 (6.3)	0.001
Fatigue	244 (22.1)	138 (20.4)	106 (24.6)	0.118
Chest pain	79 (7.1)	43 (6.4)	36 (8.4)	0.232
Diarrhea	227 (20.5)	133 (19.7)	94 (21.8)	0.402
Abdominal pain	28 (2.5)	14 (2.1)	14 (3.2)	0.243
Anorexia	198 (17.9)	105 (15.6)	93 (21.6)	0.013
Nausea or Vomiting	95 (8.6)	47 (7.0)	48 (11.1)	0.02
Myalgia	120 (10.8)	63 (9.3)	57 (13.2)	0.047
Headache	84 (7.6)	39 (5.8)	45 (10.4)	0.005
Dizziness	27 (2.4)	11 (1.6)	16 (3.7)	0.044
**Vital signs**				
Respiratory rate, breaths per minute	20.00 [19.00, 22.00]	20.00 [18.00, 20.00]	22.00 [20.00, 30.00]	<0.001
Pulse, beat per minute	82.00 [76.00, 94.00]	82.00 [76.00, 92.00]	84.00 [77.00, 95.00]	0.025
Median arterial pressure, mmHg	96.67 [88.67, 105.67]	96.67 [88.67, 105.67]	97.00 [89.33, 105.67]	0.679
percutaneous oxygen saturation, %	97.00 [95.00, 98.00]	98.00 [96.00, 99.00]	96.00 [92.00, 98.00]	<0.001
**Comorbidities**				
In-hospital death, %	58 (5.2)	13 (1.9)	45 (10.4)	<0.001
Acute respiratory distress syndrome	122 (11.0)	23 (3.4)	99 (23.0)	<0.001
Acute kidney injury	56 (5.1)	23 (3.4)	33 (7.7)	0.003
Acute heart failure	131 (13.1)	43 (7.2)	88 (21.9)	<0.001
Sepsis	105 (9.5)	29 (4.3)	76 (17.6)	<0.001
Acute liver injury, %	5 (0.5)	0 (0.0)	5 (1.2)	0.009
Hyper-glycaemia, %	665 (60.7)	446 (66.9)	219 (51.0)	<0.001
Secondary bacteria infection	23 (2.1)	7 (1.0)	16 (3.7)	0.004
**Treatment**				
Nasal cannula	1002 (90.8)	675 (100)	427 (99.1)	0.023
High flow	399 (36.1)	163 (24.1)	236 (54.8)	<0.001
Non-invasive	46 (4.2)	10 (1.5)	36 (8.4)	<0.001
Invasive	61 (5.5)	13 (1.9)	48 (11.1)	<0.001
Extracorporeal membrane oxygenation	6 (0.5)	2 (0.3)	4 (0.9)	0.216
Renal replacement therapy	40 (3.6)	9 (1.3)	31 (7.2)	<0.001
Antiviral agents	1017 (92.0)	620 (91.9)	397 (92.1)	0.91
Antibacterial agents	759 (68.6)	424 (62.8)	335 (77.7)	<0.001
Glucocorticoids	533 (48.2)	295 (43.7)	238 (55.2)	<0.001
Immunoglobulin	220 (19.9)	97 (14.4)	123 (28.5)	<0.001

*Data are median (IQR), numbers (percentages) of patients. p-values comparing Moderate and Severe+Critically ill are from χ^2^-test, Fisher's exact test, or Mann–Whitney U-test. COVID-2019, coronavirus disease 2019; The severity was staged based on the guidelines for diagnosis and treatment of COVID-19 (trial seventh edition) published by Chinese National Health Commission in February 4, 2020*.

Compared to the patients with moderate disease, the severely and critically ill patients were more likely to have fever. Symptoms including shortness of breath, myalgia, rhinorrhea, anorexia, nausea or vomiting, headache and dizziness were more common in severely and critically ill patients. Vital signs including respiratory rate and pulse were higher in severely and critically ill patients than in those with moderate disease [22 (IQR20.0-30.0) vs. 20 (IQR18.0-20.0), 84 (IQR77.0-95.0) vs. 82 (IQR76.0-92.0)], and percutaneous oxygen saturation (SpO_2_) in severely and critically ill patients was lower than in patients with moderate disease [96% (92.0–98.0) vs. 98% (96.0–99.0)]. The severely and critically ill patients were more likely to have comorbidities such as ARDS (23.0 vs. 3.4%), sepsis (17.6 vs. 4.3%), acute heart failure (21.9 vs. 7.2%), AKI (7.7 vs. 3.4%), acute liver injury (1.2 vs. 0.0%), and secondary bacterial infections (3.7 vs. 1.0%). The proportion of severely and critically ill patients requiring a high-flow nasal cannula (HFNC), NMV, IMV, CRRT, antibacterial agents, glucocorticoids, or immunoglobulins was higher than that of patients with moderate disease (Table 1).

Leukocyte and neutrophil counts in severely and critically ill patients were higher than in patients with moderate disease [6.05 × 10^9^/L (IQR4.79–8.14) vs. 5.66 × 10^9^/L (IQR4.62–7.00), 4.20 × 10^9^/L (IQR2.83–6.15) vs. 3.46 × 10^9^/L (IQR2.62–4.62)], while the lymphocyte count was lower in severely and critically ill patients than in those with moderate disease [1.07 × 10^9^/L (IQR 0.71–1.47) vs. 1.41 × 10^9^ (IQR1.04–1.83)]. D-dimer and fibrinogen levels were higher in severely and critically ill patients than in those with moderate disease [0.97 μg/ml FEU (IQR0.41–2.62) vs. 0.44 μg/ml FEU (IQR0.22–0.95), 4.71 g/L (IQR3.66–5.96) vs. 3.97 g/L (IQR3.22–5.25)]. Higher levels of serum aspartate aminotransferase, total bilirubin, direct bilirubin, albumin, alkaline phosphatase, γ-glutamyl transpeptidase, total cholesterol, and lactose dehydrogenase were more common in the severely and critically ill groups. The serum levels of creatinine kinase, high-sensitivity cardiac troponin-I (c-TnI), N-terminal pro-brain natriuretic peptide (NT-proBNP) and myoglobin were higher in severely and critically ill patients than in those with moderate disease [57.00 U/L (IQR36.00–94.50) vs. 55.00 U/L (IQR40.00–79.00), 5.70 pg/ml (IQR2.60–14.10) vs. 2.70 pg/ml (IQR1.00–6.38), 145.00 pg/ml (IQR59.00–451.00) vs. 72.00 pg/ml (IQR30.00–205.00), 46.50 ng/ml (IQR29.92–93.68) vs. 33.6 5ng/ml (IQR25.02–50.05)] (Table 2).

**Table 2:** Laboratory findings on admission in COVID-19 patients enrolled in this study.

	**Normal range**	**All** **patients**	**Grade**		***P*** **value**
			**Moderate**	**Severe+** **Critically ill**	
**Hematologic**					
Leukocyte count, ×10^9^/L	3.5–9.5	5.81 [4.68, 7.39]	5.66 [4.62, 7.00]	6.05 [4.79, 8.14]	<0.001
Neutrophil count, ×10^9^/L	1.8–6.3	3.68 [2.70, 5.18]	3.46 [2.62, 4.62]	4.20 [2.83, 6.15]	<0.001
Lymphocyte count, ×10^9^/L	1.1–3.2	1.30 [0.89, 1.70]	1.41 [1.04, 1.83]	1.07 [0.71, 1.47]	<0.001
Platelet count, ×10^9^/L	125–350	234.00 [180.00, 304.00]	235.50 [187.00, 308.75]	230.00 [170.50, 301.00]	0.027
Hemoglobin, g/L	115–150	126.00 [116.00, 137.00]	127.00 [117.00, 137.75]	126.00 [115.00, 137.00]	0.163
**Coagulation function**					
Prothrombin time, s	11.5–14.5	13.60 [13.00, 14.10]	13.40 [12.90, 14.00]	13.80 [13.20, 14.50]	<0.001
Activated partial thromboplastin time, s	29–42	38.10 [35.50, 41.10]	37.90 [35.50, 41.00]	38.15 [35.50, 41.27]	0.734
D-dimer, ug/ml FEU	<0.5	0.59 [0.26, 1.45]	0.44 [0.22, 0.95]	0.97 [0.41, 2.62]	<0.001
Fibrinogen, g/L	2.00–4.00	4.27 [3.36, 5.58]	3.97 [3.22, 5.25]	4.71 [3.66, 5.96]	<0.001
Prothrombin activity, %	75.0–125.0	93.00 [86.00, 100.00]	94.00 [87.00, 102.00]	90.00 [81.00, 98.00]	<0.001
International standardized ratio	0.80–1.20	1.05 [0.99, 1.10]	1.03 [0.98, 1.09]	1.06 [1.01, 1.14]	<0.001
**Biochemical liver function**					
Alanine aminotransferase, U/L	≤ 41	22.00 [14.00, 37.00]	21.00 [14.00, 37.00]	23.00 [14.50, 38.00]	0.133
Aspartate aminotransferase, U/L	≤ 40	23.00 [17.00, 34.00]	22.00 [17.00, 31.00]	26.00 [19.00, 39.00]	<0.001
Total bilirubin, umol/L	≤ 21.1	8.20 [6.20, 11.88]	7.90 [5.80, 11.15]	8.70 [6.55, 12.95]	<0.001
Direct bilirubin, umol/L	≤ 8.0	3.50 [2.70, 4.97]	3.40 [2.55, 4.60]	3.90 [2.80, 5.60]	<0.001
Alkaline phosphatase, U/L	35–105	36.75 [32.60, 41.10]	38.30 [34.10, 42.05]	34.30 [30.75, 38.60]	<0.001
γ-glutamyl transpeptidase, U/L	6–42	26.50 [18.00, 47.00]	25.00 [17.00, 43.00]	29.00 [19.00, 55.50]	0.002
Albumin, g/L	35.0–52.0	230.00 [164.25, 273.75]	240.50 [193.50, 279.00]	198.00 [123.25, 258.25]	<0.001
Pre-albumin, mg/L	200–400	3.81 [3.24, 4.47]	3.86 [3.33, 4.50]	3.73 [3.12, 4.41]	0.003
Total cholesterol, mmol/L	<5.18	237.50 [188.00, 311.50]	219.00 [180.00, 282.00]	279.00 [211.00, 384.00]	<0.001
lactose dehydrogenase, U/L	135–214	7041.00 [5580.00, 8452.00]	7474.00 [6083.00, 8713.00]	6416.00 [4656.25, 7722.25]	<0.001
Cholinesterase, U/L	5320–12920	5.67 [5.05, 7.06]	5.45 [4.97, 6.59]	6.06 [5.23, 7.76]	<0.001
Glucose, mM		22.00 [14.00, 37.00]	21.00 [14.00, 37.00]	23.00 [14.50, 38.00]	0.133
**Biochemical renal function**					
Creatinine, umol/L	45–84	67.00 [56.00, 82.00]	67.00 [56.00, 80.00]	69.00 [56.00, 84.00]	0.125
Blood urea nitrogen, mmol/L	1.7–8.3	4.40 [3.50, 5.70]	4.30 [3.50, 5.35]	4.60 [3.50, 6.35]	0.001
Uric acid, umol/L	142.8–339.2	265.10 [203.07, 331.00]	269.00 [212.60, 335.20]	257.00 [188.80, 324.20]	0.008
eGFR, ml/min/1.73m^2^	>90	92.25 [79.10, 101.97]	93.50 [80.25, 103.70]	90.40 [75.55, 99.30]	<0.001
Sodium, mmol/L	136–145	139.70 [137.40, 141.40]	139.90 [138.00, 141.40]	139.30 [136.20, 141.40]	0.001
Potassium, mmol/L	3.50–5.10	4.13 [3.75, 4.44]	4.17 [3.81, 4.44]	4.07 [3.69, 4.42]	0.023
Calcium, mmol/L	2.20–2.55	2.13 [2.05, 2.20]	2.15 [2.08, 2.22]	2.09 [2.01, 2.18]	<0.001
**Biochemical cardiac function**					
Creatinine kinase, U/L	≤ 190	56.00 [38.50, 84.00]	55.00 [40.00, 79.00]	57.00 [36.00, 94.50]	0.483
High–sensitivity cardiac troponin I (hs-cTnI), pg/ml	≤ 15.6	3.85 [1.00, 9.03]	2.70 [1.00, 6.38]	5.70 [2.60, 14.10]	<0.001
N-terminal pro-brain natriuretic peptide (NT-proBNP), pg/ml	<486	96.00 [37.00, 279.50]	72.00 [30.00, 205.00]	145.00 [59.00, 451.00]	<0.001
Myoglobin, ng/ml	<70	37.00 [26.78, 63.47]	33.65 [25.02, 50.25]	46.50 [29.92, 93.68]	<0.001

*Data are median (IQR), numbers (percentages) of patients. p-values comparing Moderate and Severe+Critically ill are from χ^2^-test, Fisher's exact test, or Mann–Whitney U-test. COVID-2019, coronavirus disease 2019; The severity was staged based on the guidelines for diagnosis and treatment of COVID-19 (trial fifth edition) published by Chinese National Health Commission in February 4, 2020*.

Inflammation-related indices [high-sensitivity C-reactive protein (hs-CRP), erythrocyte sedimentation rate (ESR), serum ferritin, and procalcitonin] were significantly higher in the severely and critically ill group than in the moderate group [21.15 mg/L (IQR2.92–71.57) vs. 3.65 mg/L (IQR1.00–22.08), 28.00 mm/h (IQR13.00–47.75) vs. 14.00 mm/h (IQR6.50–38.50), 638.20 μg/L(IQR326.60–1047.20) vs. 399.00 μg/L (IQR187.62–704.17), 0.08 ng/ml (IQR0.06–0.17) vs. 0.06 ng/ml(IQR0.05–0.08)]. A comparison of cytokines levels, including IL-6, IL2R, IL-8, and TNF-α, between the two groups also showed similar trends [5.79 pg/ml (IQR2.43–20.10) vs. 3.15 pg/ml (IQR1.70–7.78), 589.00 U/ml (IQR397.00–917.00) vs. 448.50 U/ml (IQR306.00–679.75), 12.60 pg/ml (IQR7.50–22.50) vs. 8.80 pg/ml (IQR5.90–15.22), 8.80 pg/ml (IQR6.30–11.50) vs. 8.00 pg/ml (IQR6.10-9.93)] (Table 3).

**Table 3:** Laboratory findings on admission in COVID-19 patients enrolled in this study.

	**Normal range**	**All** **patients**	**Moderate**	**Severe +** **Critically ill**	***P*-value**
**Inflammation-related indices**					
hs-CRP, mg/L	<10	7.25 [1.40, 41.32]	3.65 [1.00, 22.08]	21.15 [2.92, 71.57]	<0.001
ESR, mm/h	<15	20.00 [9.00, 45.00]	14.00 [6.50, 38.50]	28.00 [13.00, 47.75]	<0.001
Serum ferritin, ug/L	30–400	508.20 [254.60, 903.70]	399.00 [187.62, 704.17]	638.20 [326.60, 1047.20]	<0.001
IL-6, pg/ml	<7.0	3.74 [1.84, 11.37]	3.15 [1.70, 7.78]	5.79 [2.43, 20.10]	<0.001
IL-1β, pg/ml	<5.0	4.90 [4.90, 4.90]	4.90 [4.90, 4.90]	4.90 [4.90, 4.90]	0.673
IL2R, U/ml	223–710	496.00 [333.50, 748.00]	448.50 [306.00, 676.75]	589.00 [397.00, 917.00]	<0.001
IL-8, pg/ml	<62	10.30 [6.30, 17.90]	8.80 [5.90, 15.22]	12.60 [7.50, 22.50]	<0.001
IL-10, pg/ml	<9.1	4.90 [4.90, 4.90]	4.90 [4.90, 4.90]	4.90 [4.90, 5.10]	0.002
TNF-α, pg/ml	<8.1	8.20 [6.20, 10.60]	8.00 [6.10, 9.93]	8.80 [6.30, 11.50]	0.001
Procalcitonin, ng/ml	0.02–0.05	0.06 [0.05, 0.10]	0.06 [0.05, 0.08]	0.08 [0.06, 0.17]	<0.001

*Data are median (IQR), numbers (percentages) of patients. p-values comparing Moderate and Severe+Critically ill are from χ^2^-test, Fisher's exact test, or Mann–Whitney U-test. COVID-2019, coronavirus disease 2019; The severity was staged based on the guidelines for diagnosis and treatment of COVID-19 (trial fifth edition) published by Chinese National Health Commission in February 4, 2020*.

### Demographics and Baseline Characteristics of Severely and Critically Ill COVID-19 Patients With or Without Intubation

As shown in Table 4, 431 severely and critically ill patients were grouped according to the need for intubation. The intubated group (median age 69 years, range 44–87) was older than the group that did not require intubation (64 years, range 23–92). Fever and chronic pulmonary disease were more common in intubated patients [42 (87.5%) vs. 282 (73.6%), 9 (18.8%) vs. 30 (7.8%)]. The incidence of comorbidities including acute heart failure, sepsis, AKI, and secondary infection was higher in the intubated group than in patients that did not require intubation [41 (85.4%) vs. 47 (13.3%), 43 (89.6%) vs. 33 (8.6%), 23 (47.9%) vs. 10 (2.6%), 13 (27.1%) vs. 28 (0.8%)]. There were significant differences at baseline between the intubated patients and those not requiring intubation in terms of routine blood parameters, coagulation, liver and kidney function, myocardial enzyme spectrum, NT-proBNP, infection related indices and cytokines. The application frequency of ECMO, CRRT, antibacterial agents, glucocorticoids and immunoglobulins as significantly higher in the intubated group than that in the group that did not require intubation [4 (8.3%) vs. 0 (0.0%), 26 (54.2%) vs. 5 (1.3%), 47 (97.9%) vs. 288 (75.2), 39 (81.2%) vs. 199 (52.0%), 39 (81.2%) vs. 84 (21.9%)]. The mortality of intubated patients was significantly higher than of patients that did not require intubation (64.6 vs. 3.7%).

**Table 4:** Characteristics and treatments in Severe+Critically ill COVID-19 patients.

	**All patients** **(*N* = 431)**	**Intubation**		***P*-value**
		**Yes**	**No**	
		**(*N* = 48)**	**(*N* = 383)**	
**Demographic characteristics**				
Age- yr	65 [23–92]	69 [44–87]	64 [23–92]	0.001
Age≥ 65	218 (50.6)	31 (64.6)	187 (48.8)	0.046
Gender-Female	211 (49.0)	15 (31.2)	196 (51.2)	0.009
**Personal history**				
Smoking history	7 (1.6)	2 (4.2)	5 (1.3)	0.178
Current smoker	3 (0.7)	1 (2.1)	2 (0.5)	0.299
Former smoker	4 (0.9)	1 (2.1)	3 (0.8)	0.378
**Coexisting disorder**				
Cardiovascular disease	39 (9.0)	6 (12.5)	33 (8.6)	0.419
Hypertension	145 (33.6)	19 (39.6)	126 (32.9)	0.418
Diabetes	80 (18.6)	9 (18.8)	71 (18.5)	1
Cerebrovascular disease	17 (3.9)	4 (8.3)	13 (3.4)	0.108
Chronic pulmonary disease	39 (9.0)	9 (18.8)	30 (7.8)	0.027
Chronic kidney disease	13 (3.0)	1 (2.1)	12 (3.1)	1
Malignancy	36 (8.4)	4 (8.3)	32 (8.4)	1
Chronic liver disease	18 (4.2)	1 (2.1)	17 (4.4)	0.707
**Signs and symptoms**				
Fever	324 (75.2)	42 (87.5)	282 (73.6)	0.035
Cough	346 (80.3)	38 (79.2)	308 (80.4)	0.848
Expectoration	261 (60.6)	30 (62.5)	231 (60.3)	0.876
Shortness of breath	206 (47.8)	31 (64.6)	175 (45.7)	0.014
Pharyngalgia	44 (10.2)	5 (10.4)	39 (10.2)	1
Rhinorrhoea	27 (6.3)	0 (0.0)	27 (7.0)	0.058
Fatigue	106 (24.6)	17 (35.4)	89 (23.2)	0.075
Chest pain	36 (8.4)	3 (6.2)	33 (8.6)	0.784
Diarrhea	94 (21.8)	12 (25.0)	82 (21.4)	0.58
Abdominal pain	14 (3.2)	2 (4.2)	12 (3.1)	0.662
Anorexia	93 (21.6)	12 (25.0)	81 (21.1)	0.577
Nausea or Vomiting	48 (11.1)	4 (8.3)	44 (11.5)	0.632
Myalgia	57 (13.2)	9 (18.8)	48 (12.5)	0.256
Headache	45 (10.4)	10 (20.8)	35 (9.1)	0.021
Respiratory rate, breaths per minute	22.00 [20.00, 30.00]	22.50 [20.00, 30.00]	21.00 [20.00, 30.00]	0.452
Pulse, beat per minute	84.00 [77.00, 95.00]	90.00 [80.75, 99.00]	84.00 [76.00, 95.00]	0.021
Median arterial pressure, mmHg	97.00 [89.33, 105.67]	95.83 [87.17, 102.00]	97.33 [89.67, 105.83]	0.217
percutaneous oxygen saturation, %	96.00 [92.00, 98.00]	92.00 [87.75, 98.00]	96.00 [92.00, 98.00]	0.005
**Comorbidities**				
Acute respiratory distress syndrome	99 (23.0)	48 (100.0)	51 (13.3)	<0.001
Acute kidney injury	33 (7.7)	23 (47.9)	10 (2.6)	<0.001
Acute heart failure	88 (21.9)	41 (85.4)	47 (13.3)	<0.001
Sepsis	76 (17.6)	43 (89.6)	33 (8.6)	<0.001
Hyper-glycaemia, %	219 (51.0)	15 (31.2)	204 (53.5)	0.005
Secondary infection	16 (3.7)	13 (27.1)	3 (0.8)	<0.001
**Treatments**				
Extracorporeal membrane oxygenation	4 (0.9)	4 (8.3)	0 (0.0)	<0.001
Renal replacement therapy	31 (7.2)	26 (54.2)	5 (1.3)	<0.001
Antiviral agents	397 (92.1)	40 (83.3)	357 (93.2)	0.04
Antibacterial agents	335 (77.7)	47 (97.9)	288 (75.2)	<0.001
Glucocorticoids	238 (55.2)	39 (81.2)	199 (52.0)	<0.001
Immunoglobulin	123 (28.5)	39 (81.2)	84 (21.9)	<0.001
**Outcomes**				
In-hospital death, %	45 (10.4)	31 (64.6)	14 (3.7)	<0.001
**Hematologic tests**				
Leukocyte count, ×10^9^/L	6.05 [4.79, 8.14]	8.36 [6.16, 11.37]	5.91 [4.76, 7.90]	<0.001
Neutrophil count, ×10^9^/L	4.20 [2.83, 6.15]	7.19 [5.02, 9.34]	3.99 [2.79, 5.79]	<0.001
Lymphocyte count, ×10^9^/L	1.07 [0.71, 1.47]	0.60 [0.48, 0.88]	1.16 [0.76, 1.50]	<0.001
Platelet count, ×10^9^/L	230.00 [170.50, 301.00]	174.50 [109.50, 264.00]	235.00 [177.50, 303.00]	<0.001
Hemoglobin, g/L	126.00 [115.00, 137.00]	138.00 [118.50, 143.25]	125.00 [115.00, 135.00]	0.004
**Coagulation function**				
Prothrombin time, s	13.80 [13.20, 14.50]	15.00 [13.88, 16.10]	13.70 [13.20, 14.30]	<0.001
Activated partial thromboplastin time, s	38.15 [35.50, 41.27]	39.25 [35.82, 43.10]	38.10 [35.40, 40.90]	0.253
D-dimer, ug/ml FEU	0.97 [0.41, 2.62]	4.45 [1.54, 22.00]	0.82 [0.36, 2.10]	<0.001
Fibrinogen, g/L	4.71 [3.66, 5.96]	5.13 [4.34, 6.28]	4.64 [3.64, 5.94]	0.135
Prothrombin activity, %	90.00 [81.00, 98.00]	77.00 [67.00, 88.50]	91.00 [84.00, 98.00]	<0.001
**Biochemical liver function**				
Alanine aminotransferase, U/L	23.00 [14.50, 38.00]	31.00 [18.00, 46.25]	22.00 [14.00, 36.50]	0.017
Aspartate aminotransferase, U/L	26.00 [19.00, 39.00]	38.50 [24.75, 59.75]	25.00 [18.00, 36.00]	<0.001
Total bilirubin, umol/L	8.70 [6.55, 12.95]	11.65 [9.62, 18.98]	8.30 [6.30, 12.45]	<0.001
Albumin, g/L	34.30 [30.75, 38.60]	31.60 [29.10, 33.42]	34.90 [31.00, 39.45]	<0.001
Pre-albumin, mg/L	198.00 [123.25, 258.25]	101.00 [79.00, 140.00]	221.00 [150.50, 263.00]	<0.001
lactose dehydrogenase, U/L	279.00 [211.00, 384.00]	453.00 [318.75, 616.50]	266.00 [203.50, 348.00]	<0.001
**Biochemical renal function**				
Creatinine, umol/L	69.00 [56.00, 84.00]	81.50 [64.75, 105.50]	69.00 [56.00, 82.00]	0.001
Blood urea nitrogen, mmol/L	4.60 [3.50, 6.35]	7.65 [5.10, 10.43]	4.40 [3.40, 5.80]	<0.001
eGFR, ml/min/1.73m^2^	90.40 [75.55, 99.30]	75.45 [54.80, 94.35]	91.20 [79.25, 100.15]	<0.001
Sodium, mmol/L	139.30 [136.20, 141.40]	138.15 [134.75, 140.48]	139.40 [136.25, 141.50]	0.141
Potassium, mmol/L	4.07 [3.69, 4.42]	4.25 [3.70, 4.74]	4.05 [3.69, 4.37]	0.14
Calcium, mmol/L	2.09 [2.01, 2.18]	2.03 [1.98, 2.10]	2.10 [2.02, 2.19]	0.002
**Biochemical cardiac function**				
Creatinine kinase, U/L	57.00 [36.00, 94.50]	92.00 [40.00, 166.50]	54.00 [36.00, 82.00]	0.002
high-sensitivity cardiac troponin I (hs-cTnI), pg/ml	5.70 [2.60, 14.10]	19.25 [7.78, 215.35]	4.80 [2.30, 12.07]	<0.001
N-terminal pro-brain natriuretic peptide (NT-proBNP), pg/ml	145.00 [59.00, 451.00]	774.50 [302.75, 2718.50]	127.00 [54.00, 346.00]	<0.001
**Infection related indices**				
hs-CRP, mg/L	21.15 [2.92, 71.57]	87.60 [54.95, 141.88]	14.90 [2.50, 61.92]	<0.001
ESR, mm/h	28.00 [13.00, 47.75]	33.50 [18.25, 54.75]	26.00 [13.00, 45.75]	0.298
Serum ferritin, ug/L	638.20 [326.60, 1047.20]	1204.10 [806.35, 2127.43]	480.40 [282.40, 792.80]	<0.001
IL-6, pg/ml	5.79 [2.43, 20.10]	32.33 [16.39, 64.16]	4.81 [2.16, 13.80]	<0.001
IL-1β, pg/ml	4.90 [4.90, 4.90]	4.90 [4.90, 6.07]	4.90 [4.90, 4.90]	0.202
IL2R, U/ml	589.00 [397.00, 917.00]	964.00 [594.75, 1376.50]	563.00 [384.00, 825.50]	<0.001
IL-8, pg/ml	12.60 [7.50, 22.50]	22.05 [15.18, 38.70]	11.80 [7.10, 20.90]	<0.001
IL-10, pg/ml	4.90 [4.90, 5.10]	6.75 [4.90, 10.05]	4.90 [4.90, 4.90]	<0.001
TNF-α, pg/ml	8.80 [6.30, 11.50]	10.80 [7.98, 14.18]	8.50 [6.20, 11.15]	0.002
Procalcitonin, ng/ml	0.08 [0.06, 0.17]	0.21 [0.14, 0.41]	0.07 [0.06, 0.13]	<0.001

*Data are median (IQR), numbers (percentages) of patients. p-values comparing IMV (Invasive mechanical ventilation) and no IMV therapy are from χ^2^-test, Fisher's exact test, or Mann–Whitney U-test. COVID-2019, coronavirus disease 2019; The severity was staged based on the guidelines for diagnosis and treatment of COVID-19 (trial seventh edition) published by Chinese National Health Commission in February 4, 2020*.

### Correlation Between SpO_2_/FiO_2_ and PaO_2_/FiO_2_

**Figure 1:**
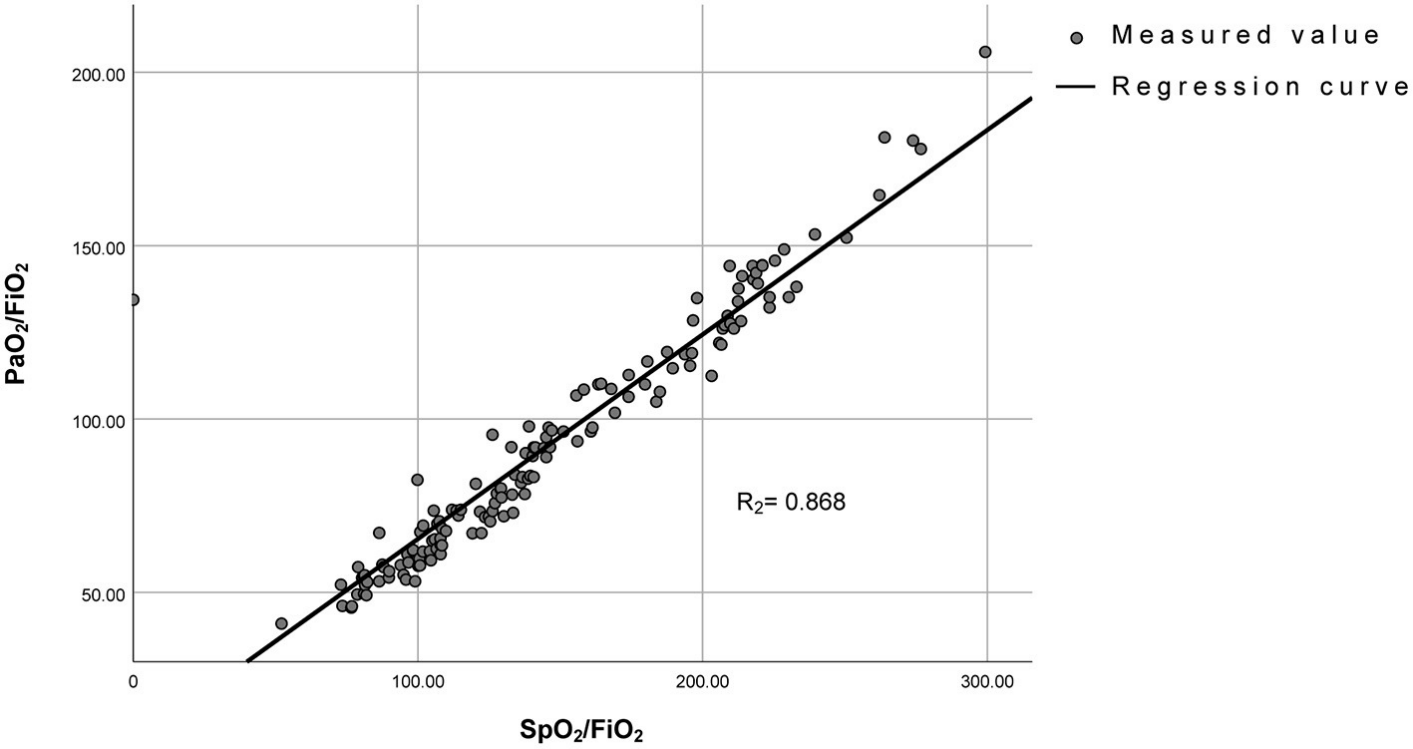
Linear correlation between PaO_2_/FiO_2_ and SpO_2_/FiO_2_. PaO_2_/FiO_2_= 0.59* SpO_2_/FiO_2_ + 6.458 (*R*^2^ = 0.868).

**Figure 2:**
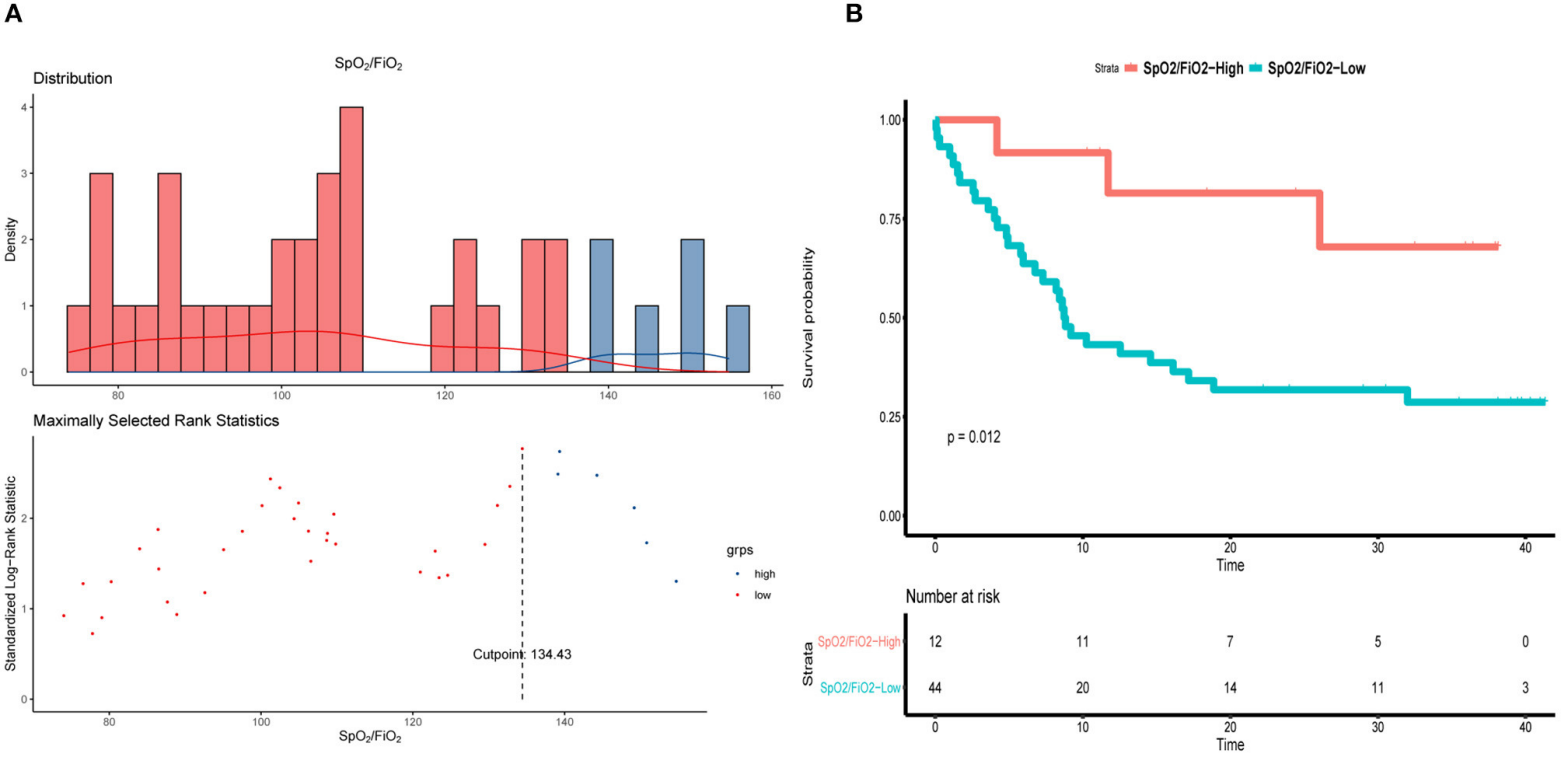
SpO_2_/FiO_2_ in patients underwent intubation during hospitalization. **(A)** Distribution of SpO_2_/FiO_2_ and cut-off value. **(B)** Kaplan–Meier curve of overall survival by using 134.43 as the cutoff for SpO_2_/FiO_2_.

Aiming to find an index that is easy to assess and can be used to monitor blood oxygenation in real-time, we evaluated the relationship between SpO_2_/FiO_2_ and PaO_2_/FiO_2_ by fitting curve analysis, which indicated that SpO_2_/FiO_2_ was positively correlated with PaO_2_/FiO_2_ (*R*^2^ = 0.8683, Figure 1). The cut-off of SpO_2_/FiO_2_ related to survival was calculated using a log-rank statistic, which yielded a value of 134.43 (Figure 2A). In addition, the optimal cutoff point for SpO_2_/FiO_2_ was also identified using ROC analysis (Figure 2B). In the survival curve analysis, the mortality of patients with SpO_2_/FiO_2_ <134.43 was significantly higher than that of patients with SpO_2_/FiO_2_ >134.43 (Figure 2B).

### Characteristics of Intubated COVID-19 Patients Stratified According to the Cut-Off Value of SpO_2_/FiO_2_

To further assess the prognostic value of the SpO_2_/FiO_2_ index, intubated COVID-19 patients were stratified according to the cut-off value of SpO_2_/FiO_2_. The values of SpO_2_/FiO_2_ were measured 0.5 h before intubation. As shown in Table 5, days from illness onset to admission differed between the low-SpO_2_/FiO_2_ (<134.43) and high-SpO_2_/FiO_2_ (>134.43) groups [18.5 days (IRQ13.30–20.00) vs. 11days (IRQ7.75–15.25)]. The mortality of patients with low SpO_2_/FiO_2_ during intubation was higher than that of patients with high SpO_2_/FiO_2_ (72.7 vs. 33.3%). Furthermore, the median arterial pressure of patients with low SpO_2_/FiO_2_ was significantly lower than that of patients with high SpO_2_/FiO_2_ [95 mmHg (IQR86.3–102.0) vs. 107.8 mmHg (IQR99.5–111.0)]. Compared to patients with high SpO_2_/FiO_2_, patients with low SpO_2_/FiO_2_ were more likely to suffer from acute heart failure [43 (97.7%) vs. 7 (58.3%)]. Cytokine levels were also analyzed, and the IL-6 levels were significantly higher in patients with low SpO_2_/FiO_2_ than in those with high SpO_2_/FiO_2_ [450.8 pg/ml (IQR182.2–5000.0) vs. 209.9 pg/ml (IQR110.6-597.7)] (Table 5).

**Table 5:** Characteristics and treatments in intubated patients who received IMV (Invasive mechanical ventilation) during hospitalization.

	**All patients** **(*N* = 56)**	**Low** **SpO_**2**_/FiO_**2**_**	**High** **SpO_**2**_/FiO_**2**_**	***P*-value**
		**(*N* = 44)**	**(*N* = 12)**	
**Demographic characteristics**				
Age- yr	69 (62.8–80.3)	70 (63.8–81)	66 (60.8–71.5)	0.174
Age≥ 65	38 (67.9)	31 (70.5)	7 (58.3)	0.32
Gender–Female	14 (25)	13 (29.5)	1 (8.3)	0.13
**Personal history**				
Smoking history	3 (5.4)	2 (4.5)	1 (8.3)	0.522
Current smoker	1 (1.8)	1 (2.3)	0	0.79
Former smoker	2 (3.6)	1 (2.3)	1 (8.3)	0.386
**Coexisting disorder**				
Cardiovascular disease	6 (10.7)	5 (11.4)	1 (8.3)	0.62
Hypertension	29 (51.8)	24 (54.5)	5 (41.7)	0.32
Diabetes	12 (21.4)	10 (22.7)	2 (16.7)	0.67
Cerebrovascular disease	4 (7.1)	4 (9.1)	0	0.37
Chronic pulmonary disease	11 (19.6)	8 (18.2)	3 (25)	0.43
Chronic kidney disease	4 (7.1)	1 (2.3)	3 (25)	0.041
Malignancy	2 (3.6)	2 (4.5)	0	0.61
Chronic liver disease	4 (7.1)	3 (6.8)	1 (8.3)	0.63
**Signs and symptoms**				
Fever (Highest body temperature, °C)				
<37.3	10 (17.9)	10 (22.7)	0	0.098
37.3–38.0	15 (26.8)	11 (25)	4 (33.3)	0.72
38.1–39.0	25 (44.6)	19 (43.2)	6 (50)	0.75
>39.0	6 (10.7)	4 (9.1)	2 (16.7)	0.60
Cough	42 (75)	32 (72.7)	10 (83.3)	0.37
Expectoration	34 (60.7)	25 (56.8)	9 (75)	0.46
Shortness of breath	22 (39.3)	14 (31.8)	8 (66.7)	0.033
Pharyngalgia	5 (8.9)	4 (9.1)	1 (8.3)	0.71
Rhinorrhoea	1 (1.8)	1 (2.3)	0	0.79
Fatigue	20 (35.7)	13 (29.5)	7 (58.3)	0.068
Chest pain	4 (7.1)	3 (6.8)	1 (8.3)	0.41
Diarrhea	12 (21.4)	12 (27.3)	0	0.038
Abdominal pain	3 (5.4)	2 (4.5)	1 (8.3)	0.26
Anorexia	14 (25)	10 (22.7)	4 (33.3)	0.34
Nausea or Vomiting	4 (7.1)	4 (9.1)	0	0.37
Myalgia	11 (19.6)	7 (15.9)	4 (33.3)	0.17
Headache	10 (17.9)	9 (20.5)	1 (8.3)	0.31
Dizziness	3 (5.4)	3 (6.8)	0	0.48
Disorders of consciousness	3 (5.4)	2 (4.5)	1 (8.3)	0.52
Respiratory rate, breaths per minute	20.5 (20–25.3)	20 (20–25)	25 (20–32.25)	0.14
Pulse, beat per minute	88 (78–97.5)	86.5 (78–96.8)	91.5 (88–97.5)	0.78
Median arterial pressure, mmHg	97.3 (87.6–107.3)	95 (86.3–102)	107.8 (99.5–111)	0.005
percutaneous oxygen saturation, %	94.5 (89.8–98)	94.5 (90.8–98)	93.5 (88.8–97)	0.85
**Comorbidities**				
Acute respiratory distress syndrome	56 (100.0)	44 (100.0)	12 (100.0)	1
Acute kidney injury	28 (50)	25 (56.8)	3 (25)	0.051
Acute heart failure	50 (89.3)	43 (97.7)	7 (58.3)	0.001
Acute liver injury	4 (7.1)	3 (6.8)	1 (8.3)	1
Cardiac injury	53 (94.6)	43 (97.7)	10 (83.3)	0.11
Hyperglycaemia	12 (21.4)	10 (22.7)	2 (16.7)	0.50
Hypoalubminemia	17 (30.4)	14 (31.8)	3 (25)	0.47
Sepsis	51 (91.1)	40 (90.9)	11 (91.7)	0.71
Secondary infection	13 (23.2)	11 (25)	2 (16.7)	0.43
**Treatments**				
Extracorporeal membrane oxygenation	5 (8.9)	4 (9.1)	1 (8.3)	0.71
Renal replacement therapy	30 (53.6)	24 (54.5)	6 (50)	0.52
Antiviral agents	55 (98.2)	43 (97.7)	12 (100)	0.79
Antibacterial agents	54 (96.4)	42 (95.5)	12 (100)	0.61
Glucocorticoids	44 (78.6)	32 (72.7)	12 (100)	0.38
Immunoglobulin	48 (85.7)	38 (86.4)	10 (83.3)	0.55
**Outcomes**				
In-hospital death, %	36 (64.3)	32 (72.7)	4 (33.3)	0.016
Days from illness onset to first outpatient visit, days	0.5 (0–6)	1 (0–6)	0 (0–2.25)	0.3
Days from illness onset to admission, days	11.5 (8–17.3)	11 (7.75–15.25)	18.5 (13.3–20)	0.018
Duration from onset of symptoms to death, days	17.3 (27–34)	24 (14.75–34)	35.5 (31.5–38.3)	0.25
Hemoglobin concentration, g/L	137.5 (120.5–145)	134.5 (119–143.3)	142.5 (130–150)	0.43
Lymphocyte count, ×10^9^/L	9.1 (6.3–14.3)	9.1 (6.3–13.8)	7.1 (5.1–16)	0.87
Platelet count, ×10^9^/L	174.5 (123–242)	176 (126.5–242)	144.5 (108–214.5)	0.62
Prothrombin time, s	17.3 (16.1–20.3)	18 (16.4–20.6)	16.8 (15.7–18.4)	0.84
Total bilirubin concentration, μmol/L	22.2 (17.9–34.6)	25.1 (17.9–38.3)	21.6 (18.8–27.7)	0.39
Direct bilirubin concentration, umol/L	5.8 (4.3–9.7)	5.8 (4.0–9.7)		0.98
Serum creatinine concentration, μmol/L	136.5 (100.5–203.5)	146.5 (108–209)	106 (75.2–144)	0.80
Lactate concentration, mmol/L	3.66 (2.93–4.15)	4.0 (3.5–6.4)	3.2 (2.8–3.9)	0.077
hs-CRP, mg/L	80.6 (41.5–125.6)	102.3 (47.3–142.4)	59 (24.1–77.9)	0.21
ESR, mm/h	30 (14–54.8)	29 (14–62.3)	40.5 (11.3–53.8)	0.70
Serum ferritin, ug/L	1,678 (1,042–3,300)	1,182 (1,046–3,896)	1,678 (675–2,713)	0.56
Interleukin−6, pg/ml	379 (144.8–3,723)	450.8 (182.2–5,000)	209.9 (110.6–597.7)	0.015
Interleukin-2R, U/mL	1,610 (1080.3–3049.3)	1770.5 (1,173–3125.3)	1,036 (816.3–1691.3)	0.32
D-dimer, ug/ml FEU	3.4 (1.3–19.5)	3.1 (1.3–17.5)	12.3 (0.6–21.0)	0.28
Fibrinogen, g/L	5.14 (3.96–6.41)	5.30 (3.96–6.77)	4.6 (3.4–5.3)	0.11
Prothrombin activity, %	77.5 (67–90.5)	76 (67–90.5)	85 (77–95.3)	0.29
Alkaline phosphatase, U/L	68.5 (62.3–88.3)	67.5 (61.3–85.8)	81 (67.3–92.8)	0.89
lactose dehydrogenase, U/L	461.5 (295–600.8)	464 (304.8–645.5)	403.5 (255.8–575.5)	0.48
Cholinesterase, U/L	5,192 (4,149–6,027)	4,918 (4,091–5,861)	5,740 (4,175–6,528)	0.31
Blood urea nitrogen, mmol/L	7.1 (4.9–10.1)	7.6 (4.9–10.1)	6.1 (4.6–9.7)	0.65
uric acid, umol/L	266.1 (179.1–348.5)	262.5 (179.1–354)	275.1 (176.1–333.2)	0.81
eGFR, ml/min/1.73m^2^	76.1 (58.8–93.5)	78.2 (58.8–91.5)	71.1 (53.3–96.3)	0.89

*Cut-off value: SpO_2_/FiO_2_ = 134.43*.

*Data are median (IQR), numbers (percentages) of patients. p-values comparing SpO_2_/FiO_2_ high and low are from χ^2^-test, Fisher's exact test, or Mann–Whitney U-test. COVID-2019, coronavirus disease 2019; The severity was staged based on the guidelines for diagnosis and treatment of COVID-19 (trial seventh edition) published by Chinese National Health Commission in February 4, 2020*.

### Characteristics of Patients Stratified According to Glucocorticoid/CRRT /Anticoagulation Therapy

Patients were treated with glucocorticoids according to the protocols in the guidelines and references (12, 16, 17). Of the 431 severely and critically ill patients, 238 received glucocorticoid treatment (55.2%), while 47 of the 61 intubated patients received glucocorticoid treatment (77.0%).

Patients with glucocorticoid therapy low lymphocytes at baseline, higher infection markers and cytokines, as well as higher incidence of comorbidities such as sepsis, acute heart failure, AKI, and secondary infection. Moreover, the proportion of patients receiving CRRT and immunoglobulin treatment among those receiving glucocorticoid treatment was also higher.

Of the 431 severely and critically ill patients, 31 (7.2%) were treated with CRRT, while 33 of the 61 intubated patients (54.1%) received CRRT. At baseline, patients treated with CRRT had high leukocyte and neutrophil counts, creatinine, cytokines, infection indicators, and ferritin. The proportion of patients administered glucocorticoids, immunoglobulins and ECMO among those receiving CRRT was also higher.

Anticoagulation therapy was used in 160 of the 431 severely and critically ill patients (37.1%). For patients with severe and critical COVID-19, risk assessment scales such as Welles's score can be used to prevent VTE. Medium- and high-risk patients can be subcutaneously injected with a half-dose of low-molecular-weight heparin once every 12 h or subcutaneously injected with one dose of low-molecular-weight heparin once a day for prevention. Of the 61 intubated patients, 60 received anticoagulation therapy (98.4%). Patients who required anticoagulation therapy had the baseline characteristics of higher levels of D-dimer, potassium, creatinine and blood urea nitrogen, as well as higher incidence of comorbidities such as sepsis, AKI, and acute heart failure (Supplementary Tables 1–4).

As detailed in Supplementary Table 5, 6 patients were treated with ECMO and five survived. Indications for implementing ECMO in COVID-19 patients include age < 70 years old, without known severe brain injury, unrecoverable heart damage, or uncontrollable hemorrhaging. For COVID-19 patients and ARDS, if the hypoxia is still not relieved after the mechanical ventilation settings have been optimized [low tidal volume < 6 mL/kg (ideal body weight) and airway plateau pressure < 30 cmH_2_O, PEEP ≥10 cmH_2_O, FiO_2_ ≥ 0.8], and prone positioning was applied for at least 12 h per day, ECMO should be considered as early as possible.

## Discussion

In this study, we analyzed the clinical data of 1,106 COVID-19 patients admitted to the Optical Valley Branch of Tongji Hospital between 9 February and 9 March, 2020. In the absence of effective antiviral drugs and vaccines, we adopted measures to detect potential severe cases and provide early intervention, multidisciplinary collaboration and comprehensive treatment, including improved oxygenation, multi-organ function support, maintaining water and electrolyte balance, restrictive liquid management, etc. Due to our efforts, the mortality of severely and critically ill patients could be reduced to 10.4%, and that of critically ill patients was 39.6%. Analyzing the applied treatments in this designated specialized hospital may provide crucial clues for understanding the strategy that determined the low mortality of critical COVID-19 patients.

Consistent with previous studies of COVID-19 patients in the United States and Italy, severely, critically ill and deceased patients had a higher incidence of preexisting conditions such as hypertension, diabetes, and chronic pulmonary disease (2, 5, 7–9, 18). Our results showed that the severely and critically ill patients were older, had more severe respiratory distress, lower lymphocyte counts, as well as higher levels of inflammatory indicators and cytokines. Patients with chronic pulmonary disease, hypertension and diabetes had a higher tendency to progress to severe COVID-19. Moreover, comorbidities such as sepsis, myocardial injury, heart failure, acute liver and kidney injury, or vascular embolism were significantly more likely to occur in severely and critically ill patients, with potentially fatal outcomes. According to these results, treatment for multi-organ dysfunction, including intubation, glucocorticoid treatment, CRRT, anticoagulation therapy and ECMO should be emphasized in reducing the mortality of severely and critically ill COVID-19 patients.

Since immune-cell infiltration, diffuse alveolar damage and small airway blockage by mucus plugs all contribute to the development of COVID-19 (19, 20), the severely and critically ill patients suffered from sustained and severe hypoxemia, resulting in a rapid deterioration of the patients' condition, and even sudden death. Therefore, it is of great importance to evaluate the level of hypoxia and rectify hypoxemia in time. Although the oxygenation index was applied in assessment of hypoxemia (12, 21), PaO_2_ requires an invasive operation and cannot be monitored in real time. In comparison, the SpO_2_/FiO_2_ measurement addressed in the WHO COVID-19 guidance is non-invasive, and the screening and monitoring of SpO_2_/FiO_2_ is more flexible when the medical practitioners have to wear full protective gear (13, 21). It was also defined by the Kigali modification of the Berlin definition and showed a correlation with the diagnosis of ARDS (22). Importantly, our results demonstrated that SpO_2_/FiO_2_ showed a positive relationship with PaO_2_/FiO_2_, indicating that SpO_2_/FiO_2_ can potentially be used as an alternative index for hypoxia. Interestingly, it seems a better correlation of SpO_2_/FiO_2_ and PaO_2_/FiO_2_ in previous study reported by Bilan et al. (23) compared to our study, which may be due to differences of sample sizes and characteristics of ARDS caused by COVID-19. Then, we calculated 134.43 as a cut-off value of SpO_2_/FiO_2_ according to the prognosis of intubated patients. When the intubated patients were stratified according to the cut-off value of SpO_2_/FiO_2_, we further found that the mortality of patients with SpO_2_/FiO_2_ <134.43 during intubation (72.7%) was much higher than that of patients with SpO_2_/FiO_2_>134.43 (33.3%), suggesting the degree of hypoxemia was correlated with mortality of intubated patients. Considering that no specific values of oxygenation index for evaluating the timing of intubation were reported in previous studies (13, 21, 24) our results provide an real-time index for early warning and timely mechanical ventilation, which might improve the outcomes to some extent.

There is increasing evidence that the rapid deterioration of critically ill COVID-19 patients may be caused by a cytokine storm characterized by explosive and potentially fatal hyper-cytokinemia and multiple organ failure, especially involving the lungs (2, 25). Previous clinical studies reported that high cytokine levels are a risk factor for mortality in critically ill COVID-19 patients (2, 18). Therefore, we analyzed the cytokine levels of intubated patients stratified according to the cut-off value of SpO_2_/FiO_2_. Interestingly, the levels of cytokines, and especially IL-6, in patients with low SpO_2_/FiO_2_ were significantly higher than in the high SpO_2_/FiO_2_ group, indicating that cytokine levels may be related to the degree of hypoxia and may also potentially offer a timely warning sign.

Glucocorticoids and CRRT are well-established as effective treatments against runaway inflammation and cytokine storms. However, the use of glucocorticoids in COVID-19 remains controversial (26). Dequin et al. reported that low-dose hydrocortisone did not significantly reduce treatment failure at day 21 compared with the placebo group (27). In contrast, Sterne et al. reported that systemic administration of corticosteroids was associated with lower 28-day all-cause mortality compared with usual care or placebo (28), while Horby et al. reported that the use of dexamethasone resulted in lower 28-day mortality among those who were receiving either invasive mechanical ventilation or oxygen alone at randomization, but not among those receiving no respiratory support (29). Also, Derek et al. reported that treatment with a 7-day fixed-dose course of hydrocortisone or shock-dependent dosing of hydrocortisone resulted in 93 and 80% better odds of not requiring organ support within 21 days (30). Indeed, benefits of glucocorticoid therapy and decreased cytokine levels were observed in some severely and critically ill patients in our study. Importantly, there seems to be a downward trend in the mortality of intubated patients with glucocorticoid treatment. Our study was consistent with current reports at least to a certain extent. In particular, the proportion of glucocorticoid usage in severely and critically ill patients was higher than in other studies from the same period (4, 5). Thus, our results showed that a high proportion of glucocorticoid treatment may be correlated with lower mortality of severely and critically ill patients. Current studies recommend that glucocorticoid therapy should be given at an appropriate dose and course at the right time (16, 17). Therefore, the timing of glucocorticoid treatment and the COVID-19 patient's own condition can determine the prognosis to a certain extent, but multi-center, random, double-blind studies with larger cohorts may be required in the future. At least but not last, a part of patients with higher cytokines, and progressive deterioration of SpO_2_/FiO_2_ may have benefited from glucocorticoid treatment.

There is accumulating evidence that CRRT is associated with lower mortality in patients with sepsis. Moreover, the removal of endotoxins and cytokines by CRRT could improve the prognosis of patients with ARDS (31). Since severe ARDS is the fundamental pathophysiology of severe viral pneumonia, CRRT was also performed in a proportion of the intubated COVID-19 patients in our cohort, which was higher than that in previously published studies (5, 32). Continuously elevated cytokine levels are regarded as announcing the onset of a cytokine storm (5). Once started, hyper-inflammation can trigger a cascade reaction leading to multiple organ failure (33). Our study showed a large increase in the expression of inflammatory makers in the intubated COVID-19 patients, and cytokine removal by CRRT was linked with a favorable prognosis. Hence, CRRT should be considered as an adjunct therapy for early treatment of critically ill COVID-19 patients, especially those with hyper-inflammation.

Although the incidence of venous thromboembolism (VTE) events in COVID-19 patients is unknown, the relationship between pneumonia and VTE is well-described (34). The activated leukocytes and cellular adhesion molecules on the vein walls contribute to the development of VTE (35). For example, H1N1 ARDS patients had high risk for pulmonary embolism and VTE (36). Given that ARDS occurred in more than half of the critically ill COVID-19 patients (5), we concluded that anticoagulation therapy should be considered in patients at high risk of trombosis. In fact, anticoagulation therapy was applied in 37.1% (160/431) of the severely and critically ill patients, as well as practically all intubated patients (60/61). There were 3 cases of mild upper gastrointestinal hemorrhage in our study. However, fatal intracranial hemorrhage was also found to be connected with the use of anticoagulants (36). The prevention of pulmonary embolism and VTE should therefore be weighed against the risks of hemorrhagic complications.

ECMO was also applied in our study. Five of the six patients who received ECMO survived until the end of the follow-up period. Early initiation of ECMO was associated with better outcomes. Although this sample was small, and the specific baseline characteristics as well as the disease courses were different, it raises concerns about potential benefits of ECMO therapy for critically ill COVID-19 patients.

Our study has several limitations. Firstly, the representativeness of study may be limited by its single-center nature and resulting data bias. Secondly, due to the retrospective nature of the study, not all laboratory indicators were available for all patients, including lactate, lymphocyte subsets, etc. Thirdly, interpretation of our result might be limited by the sample size. Finally, retrospective and observational study cannot make causal relationship between treatments and outcome. Therefore, further studies are needed to provide a better understanding of treatment options and mortality of COVID-19 patients, which may help guide efforts aimed at reducing the mortality.

In summary, we recommend the real-time tracking of early warning signs, multidisciplinary collaboration, multi-organ function support and personalized treatment, which may play key role in the prognosis of severely and critically ill COVID-19 patients.

## Data Availability Statement

The data that support the findings of this study are available from the corresponding author upon reasonable request.

## Ethics Statement

This study was approved by the Ethics Commission of Tongji hospital (Approval No.: TJ-IRB20200334), and the written informed consent requirement was waived for anonymized data in view of the rapidly emerging infectious disease.

## Author Contributions

JL, HW, and ZT made substantial contributions to the study concept and design. ZD, HF, YR, BL, JJ, NZ, FH, and YL collected the epidemiological and clinical data. ZD, ZW, HF, DW, BZ, GX, JZ, and GL summarized and analyzed all data. WZ, HZ, YL, ZL, YR, and ZD drafted the manuscript. JL, HW, ZT, HZ, and WZ revised the final manuscript. All authors contributed to the article and approved the submitted version.

## Conflict of Interest

The authors declare that the research was conducted in the absence of any commercial or financial relationships that could be construed as a potential conflict of interest.
