# Supplementary material for: Optimizing Management to Reduce the Mortality of COVID-19: Experience From a Designated Hospital for Severely and Critically Ill Patients in China
Source: Front Med (Lausanne). 2021 Mar 10;8:582764. doi: 10.3389/fmed.2021.582764 (PMC7987780; doi:10.3389/fmed.2021.582764)
Supplement: Supplementary file 1 [file Table_1.DOCX]

**Supplemental Table 1. Characteristics and Treatments in Severe+Critically ill COVID-19 patients.**

|  | **All patients** | **Glucocorticoid therapy** | | **P Value** | | **CRRT** | | **P Value** |
| --- | --- | --- | --- | --- | --- | --- | --- | --- |
|  |  | **Yes** | **No** | |  | **Yes** | **No** |  |
|  | **(N=431)** | **(N=238)** | **(N=193)** | |  | **(N=31)** | **(N=400)** |  |
| **Demographic characteristics** |  |  |  | |  |  |  |  |
| **Age- yr** | 65 [23-92] | 66 [23-92] | 64 [23-86] | | 0.014 | 71 [44-86] | 64 [23-92] | 0.002 |
| **Age≥ 65** | 218 (50.6) | 129 (54.2) | 89 (46.1) | | 0.1 | 22 (71.0) | 196 (49.0) | 0.024 |
| **Gender-Female** | 211 (49.0) | 113 (47.5) | 98 (50.8) | | 0.5 | 11 (35.5) | 200 (50.0) | 0.138 |
| **Personal history** |  |  |  | |  |  |  |  |
| **Smoking history** | 7 (1.6) | 5 (2.1) | 2 (1.0) | | 0.467 | 1 (3.2) | 6 (1.5) | 0.409 |
| **Current smoker** | 3 (0.7) | 1 (0.4) | 2 (1.0) | | 0.589 | 1 (3.2) | 2 (0.5) | 0.201 |
| **Former smoker** | 4 (0.9) | 4 (1.7) | 0 (0.0) | | 0.131 | 0 (0.0) | 4 (1.0) | 1 |
| **Coexisting disorder** |  |  |  | |  |  |  |  |
| **Cardiovascular disease** | 39 (9.0) | 21 (8.8) | 18 (9.3) | | 0.867 | 3 (9.7) | 36 (9.0) | 0.752 |
| **Hypertension** | 145 (33.6) | 82 (34.5) | 63 (32.6) | | 0.759 | 14 (45.2) | 131 (32.8) | 0.17 |
| **Diabetes** | 80 (18.6) | 44 (18.5) | 36 (18.7) | | 1 | 6 (19.4) | 74 (18.5) | 0.815 |
| **Cerebrovascular disease** | 17 (3.9) | 12 (5.0) | 5 (2.6) | | 0.222 | 0 (0.0) | 17 (4.2) | 0.624 |
| **Chronic pulmonary disease** | 39 (9.0) | 28 (11.8) | 11 (5.7) | | 0.042 | 6 (19.4) | 33 (8.2) | 0.05 |
| **Chronic kidney disease** | 13 (3.0) | 11 (4.6) | 2 (1.0) | | 0.044 | 2 (6.5) | 11 (2.8) | 0.238 |
| **Chronic liver disease** | 36 (8.4) | 19 (8.0) | 17 (8.8) | | 0.861 | 4 (12.9) | 32 (8.0) | 0.314 |
| **Malignancy** | 18 (4.2) | 9 (3.8) | 9 (4.7) | | 0.809 | 1 (3.2) | 17 (4.2) | 1 |
| **Signs and symptoms** |  |  |  | |  |  |  |  |
| **Fever** | 324 (75.2) | 187 (78.6) | 137 (71.0) | | 0.074 | 27 (87.1) | 297 (74.2) | 0.133 |
| **Cough** | 346 (80.3) | 188 (79.0) | 158 (81.9) | | 0.468 | 27 (87.1) | 319 (79.8) | 0.481 |
| **Expectoration** | 261 (60.6) | 137 (57.6) | 124 (64.2) | | 0.166 | 23 (74.2) | 238 (59.5) | 0.128 |
| **Shortness of breath** | 206 (47.8) | 115 (48.3) | 91 (47.2) | | 0.846 | 22 (71.0) | 184 (46.0) | 0.009 |
| **Pharyngalgia** | 44 (10.2) | 22 (9.2) | 22 (11.4) | | 0.523 | 4 (12.9) | 40 (10.0) | 0.542 |
| **Rhinorrhoea** | 27 (6.3) | 11 (4.6) | 16 (8.3) | | 0.161 | 0 (0.0) | 27 (6.8) | 0.244 |
| **Fatigue** | 106 (24.6) | 63 (26.5) | 43 (22.3) | | 0.368 | 13 (41.9) | 93 (23.2) | 0.029 |
| **Chest pain** | 36 (8.4) | 20 (8.4) | 16 (8.3) | | 1 | 3 (9.7) | 33 (8.2) | 0.735 |
| **Diarrhea** | 94 (21.8) | 47 (19.7) | 47 (24.4) | | 0.291 | 6 (19.4) | 88 (22.0) | 0.825 |
| **Abdominal pain** | 14 (3.2) | 7 (2.9) | 7 (3.6) | | 0.787 | 2 (6.5) | 12 (3.0) | 0.266 |
| **Anorexia** | 93 (21.6) | 56 (23.5) | 37 (19.2) | | 0.291 | 7 (22.6) | 86 (21.5) | 0.824 |
| **Nausea or Vomiting** | 48 (11.1) | 29 (12.2) | 19 (9.8) | | 0.538 | 4 (12.9) | 44 (11.0) | 0.765 |
| **Myalgia** | 57 (13.2) | 33 (13.9) | 24 (12.4) | | 0.775 | 7 (22.6) | 50 (12.5) | 0.162 |
| **Headache** | 45 (10.4) | 28 (11.8) | 17 (8.8) | | 0.345 | 7 (22.6) | 38 (9.5) | 0.032 |
| **Respiratory rate, breaths per minute** | 22.00 [20.00, 30.00] | 21.00 [20.00, 30.00] | 22.00 [20.00, 30.00] | | 0.916 | 23.00 [20.00, 30.00] | 21.00 [20.00, 30.00] | 0.371 |
| **Pulse, beat per minute** | 84.00 [77.00, 95.00] | 86.00 [77.00, 96.75] | 82.00 [77.00, 92.00] | | 0.126 | 90.00 [78.50, 99.50] | 84.00 [77.00, 95.00] | 0.088 |
| **Median arterial pressure, mmHg** | 97.00 [89.33, 105.67] | 98.50 [89.67, 106.00] | 95.33 [88.33, 105.33] | | 0.126 | 94.33 [86.83, 100.67] | 97.33 [89.67, 105.75] | 0.068 |
| **percutaneous oxygen saturation, %** | 96.00 [92.00, 98.00] | 95.00 [92.00, 98.00] | 96.00 [92.00, 98.00] | | 0.004 | 92.00 [87.00, 97.00] | 96.00 [92.00, 98.00] | 0.012 |
| **Comorbidities** |  |  |  | |  |  |  |  |
| **Acute respiratory distress syndrome** | 99 (23.0) | 78 (32.8) | 21 (10.9) | | <0.001 | 29 (93.5) | 70 (17.5) | <0.001 |
| **Acute kidney injury** | 33 (7.7) | 24 (10.1) | 9 (4.7) | | 0.044 | 16 (51.6) | 17 (4.2) | <0.001 |
| **Acute heart failure** | 88 (21.9) | 67 (29.4) | 21 (12.1) | | <0.001 | 26 (83.9) | 62 (16.8) | <0.001 |
| **Sepsis** | 76 (17.6) | 61 (25.6) | 15 (7.8) | | <0.001 | 25 (80.6) | 51 (12.8) | <0.001 |
| **Hyper-glycaemia, %** | 219 (51.0) | 110 (46.6) | 109 (56.5) | | 0.052 | 7 (22.6) | 212 (53.3) | 0.001 |
| **Secondary Infection** | 16 (3.7) | 14 (5.9) | 2 (1.0) | | 0.009 | 8 (25.8) | 8 (2.0) | <0.001 |
| **Treatments** |  |  |  | |  |  |  |  |
| **Extracorporeal membrane oxygenation** | 4 (0.9) | 4 (1.7) | 0 (0.0) | | 0.131 | 4 (12.9) | 0 (0.0) | <0.001 |
| **Renal replacement therapy** | 31 (7.2) | 24 (10.1) | 7 (3.6) | | 0.014 | 31 (100.0) | 0 (0.0) | <0.001 |
| **Antiviral agents** | 397 (92.1) | 217 (91.2) | 180 (93.3) | | 0.476 | 26 (83.9) | 371 (92.8) | 0.086 |
| **Antibacterial agents** | 335 (77.7) | 205 (86.1) | 130 (67.4) | | <0.001 | 31 (100.0) | 304 (76.0) | <0.001 |
| **Glucocorticoids** | 238 (55.2) | 238 (100.0) | 0 (0.0) | | <0.001 | 24 (77.4) | 214 (53.5) | 0.014 |
| **Immunoglobulin** | 123 (28.5) | 89 (37.4) | 34 (17.6) | | <0.001 | 26 (83.9) | 97 (24.2) | <0.001 |
| **Hematologic tests** |  |  |  | |  |  |  |  |
| **Leukocyte count, ×10^9^/L** | 6.05 [4.79, 8.14] | 6.37 [4.87, 8.66] | 5.88 [4.76, 7.56] | | 0.032 | 8.66 [4.51, 13.16] | 5.98 [4.80, 7.99] | 0.058 |
| **Neutrophil count, ×10^9^/L** | 4.20 [2.83, 6.15] | 4.56 [2.99, 7.08] | 3.81 [2.66, 5.68] | | 0.004 | 7.59 [2.96, 11.62] | 4.12 [2.83, 5.98] | 0.007 |
| **Lymphocyte count, ×10^9^/L** | 1.07 [0.71, 1.47] | 0.97 [0.62, 1.35] | 1.21 [0.85, 1.56] | | <0.001 | 0.59 [0.48, 0.82] | 1.14 [0.76, 1.49] | <0.001 |
| **Platelet count, ×10^9^/L** | 230.00 [170.50, 301.00] | 226.00 [164.25, 301.00] | 239.00 [179.00, 297.00] | | 0.143 | 152.00 [102.50, 211.00] | 235.00 [176.75, 304.00] | <0.001 |
| **Hemoglobin, g/L** | 126.00 [115.00, 137.00] | 125.00 [114.25, 137.00] | 127.00 [116.00, 136.00] | | 0.509 | 133.00 [110.00, 141.50] | 125.00 [115.75, 136.00] | 0.404 |
| **Coagulation function** |  |  |  | |  |  |  |  |
| **Prothrombin time, s** | 13.80 [13.20, 14.50] | 13.90 [13.20, 14.70] | 13.70 [13.20, 14.20] | | 0.039 | 15.00 [13.90, 16.40] | 13.70 [13.20, 14.35] | <0.001 |
| **Activated partial thromboplastin time, s** | 38.15 [35.50, 41.27] | 38.45 [35.40, 42.27] | 37.65 [35.60, 40.35] | | 0.206 | 39.70 [35.85, 43.20] | 38.10 [35.40, 40.95] | 0.193 |
| **D-dimer, ug/ml FEU** | 0.97 [0.41, 2.62] | 1.16 [0.48, 2.80] | 0.82 [0.31, 2.21] | | 0.006 | 4.59 [1.66, 20.55] | 0.88 [0.39, 2.27] | <0.001 |
| **Fibrinogen, g/L** | 4.71 [3.66, 5.96] | 4.89 [3.84, 5.87] | 4.34 [3.42, 5.97] | | 0.044 | 4.86 [3.36, 5.78] | 4.71 [3.66, 5.96] | 0.641 |
| **Prothrombin activity, %** | 90.00 [81.00, 98.00] | 89.00 [79.00, 98.00] | 91.00 [85.00, 98.00] | | 0.032 | 76.00 [65.50, 88.50] | 91.00 [83.00, 98.00] | <0.001 |
| **Biochemical liver function** |  |  |  | |  |  |  |  |
| **Alanine aminotransferase, U/L** | 23.00 [14.50, 38.00] | 24.00 [15.00, 38.75] | 21.00 [14.00, 37.00] | | 0.222 | 32.00 [19.50, 51.00] | 22.00 [14.00, 37.00] | 0.011 |
| **Aspartate aminotransferase, U/L** | 26.00 [19.00, 39.00] | 26.00 [19.00, 39.75] | 25.00 [18.00, 38.00] | | 0.246 | 42.00 [31.50, 65.00] | 25.00 [18.00, 37.25] | <0.001 |
| **Total bilirubin, umol/L** | 8.70 [6.55, 12.95] | 9.20 [7.12, 13.17] | 8.00 [6.20, 12.10] | | 0.005 | 11.80 [8.05, 17.25] | 8.50 [6.40, 12.62] | 0.009 |
| **Albumin, g/L** | 34.30 [30.75, 38.60] | 33.30 [29.92, 37.48] | 36.00 [32.40, 40.40] | | <0.001 | 31.10 [28.65, 33.35] | 34.80 [31.00, 39.32] | <0.001 |
| **Pre-albumin, mg/L** | 198.00 [123.25, 258.25] | 189.00 [98.00, 253.00] | 221.00 [140.00, 273.00] | | 0.018 | 124.00 [79.00, 140.00] | 210.00 [131.50, 260.50] | <0.001 |
| **lactose dehydrogenase, U/L** | 279.00 [211.00, 384.00] | 300.00 [222.25, 409.50] | 257.00 [190.00, 333.00] | | <0.001 | 460.00 [366.00, 596.50] | 267.00 [207.00, 357.25] | <0.001 |
| **Biochemical renal function** |  |  |  | |  |  |  |  |
| **Creatinine, umol/L** | 69.00 [56.00, 84.00] | 69.00 [56.00, 84.75] | 70.00 [56.00, 83.00] | | 0.945 | 98.00 [68.50, 132.50] | 68.50 [56.00, 82.00] | <0.001 |
| **Blood urea nitrogen, mmol/L** | 4.60 [3.50, 6.35] | 4.70 [3.62, 6.68] | 4.50 [3.40, 5.90] | | 0.075 | 8.00 [5.00, 12.10] | 4.50 [3.50, 5.90] | <0.001 |
| **eGFR, ml/min/1.73m^2^** | 90.40 [75.55, 99.30] | 89.50 [74.28, 97.80] | 91.60 [77.50, 100.70] | | 0.118 | 66.20 [43.20, 81.50] | 91.20 [79.10, 99.97] | <0.001 |
| **Sodium, mmol/L** | 139.30 [136.20, 141.40] | 139.00 [135.72, 141.20] | 139.50 [136.80, 141.60] | | 0.166 | 136.90 [134.45, 140.50] | 139.30 [136.47, 141.50] | 0.024 |
| **Potassium, mmol/L** | 4.07 [3.69, 4.42] | 4.02 [3.61, 4.37] | 4.13 [3.75, 4.48] | | 0.036 | 4.35 [3.56, 4.79] | 4.05 [3.69, 4.37] | 0.081 |
| **Calcium, mmol/L** | 2.09 [2.01, 2.18] | 2.06 [1.99, 2.16] | 2.12 [2.04, 2.19] | | <0.001 | 2.00 [1.96, 2.05] | 2.10 [2.02, 2.19] | <0.001 |
| **Biochemical cardiac function** |  |  |  | |  |  |  |  |
| **Creatinine kinase, U/L** | 57.00 [36.00, 94.50] | 57.00 [38.00, 106.00] | 57.00 [35.00, 87.00] | | 0.502 | 73.50 [36.00, 339.50] | 56.00 [36.00, 89.25] | 0.095 |
| **high-sensitivity cardiac troponin I (hs-cTnI), pg/ml** | 5.70 [2.60, 14.10] | 6.60 [2.90, 16.60] | 4.60 [2.00, 11.40] | | 0.006 | 25.30 [6.25, 215.15] | 5.20 [2.45, 12.50] | <0.001 |
| **N-terminal pro-brain natriuretic peptide**  **(NT-****proBNP), pg/ml** | 145.00 [59.00, 451.00] | 165.50 [72.50, 623.75] | 125.00 [41.00, 303.00] | | 0.001 | 570.00 [316.50, 2853.00] | 129.50 [56.25, 378.00] | <0.001 |
| **Infection related indices** |  |  |  | |  |  |  |  |
| **hs-CRP, mg/L** | 21.15 [2.92, 71.57] | 32.40 [6.50, 78.30] | 9.20 [2.20, 58.00] | | <0.001 | 93.30 [62.20, 116.60] | 17.80 [2.60, 63.50] | <0.001 |
| **ESR, mm/h** | 28.00 [13.00, 47.75] | 35.50 [19.00, 54.00] | 19.00 [11.00, 33.50] | | 0.008 | 35.00 [10.00, 54.00] | 27.00 [13.00, 46.50] | 0.848 |
| **Serum ferritin, ug/L** | 638.20 [326.60, 1047.20] | 765.30 [432.55, 1413.05] | 406.45 [246.35, 730.70] | | 0.001 | 1413.25 [877.28, 2202.53] | 544.60 [301.00, 878.15] | <0.001 |
| **IL-6, pg/ml** | 5.79 [2.43, 20.10] | 8.55 [3.12, 27.46] | 3.85 [1.84, 10.96] | | <0.001 | 33.97 [18.39, 65.76] | 5.06 [2.21, 16.35] | <0.001 |
| **IL-1β, pg/ml** | 4.90 [4.90, 4.90] | 4.90 [4.90, 4.90] | 4.90 [4.90, 4.90] | | 0.576 | 4.90 [4.90, 6.65] | 4.90 [4.90, 4.90] | 0.115 |
| **IL2R, U/ml** | 589.00 [397.00, 917.00] | 666.00 [436.00, 1040.00] | 510.00 [362.00, 737.50] | | <0.001 | 1055.00 [634.00, 1483.50] | 571.00 [386.00, 851.50] | <0.001 |
| **IL-8, pg/ml** | 12.60 [7.50, 22.50] | 13.95 [8.03, 26.17] | 11.10 [6.95, 17.80] | | 0.001 | 22.20 [14.40, 39.65] | 12.30 [7.23, 21.98] | <0.001 |
| **IL-10, pg/ml** | 4.90 [4.90, 5.10] | 4.90 [4.90, 6.20] | 4.90 [4.90, 4.90] | | 0.003 | 6.40 [4.90, 10.30] | 4.90 [4.90, 4.90] | <0.001 |
| **TNF-α, pg/ml** | 8.80 [6.30, 11.50] | 9.30 [6.82, 11.97] | 7.90 [5.85, 10.85] | | 0.002 | 11.00 [8.35, 14.05] | 8.60 [6.23, 11.20] | 0.001 |
| **Procalcitonin, ng/ml** | 0.08 [0.06, 0.17] | 0.09 [0.07, 0.22] | 0.07 [0.05, 0.12] | | <0.001 | 0.28 [0.15, 0.60] | 0.08 [0.06, 0.14] | <0.001 |

Data are median (IQR), numbers (percentages) of patients. p values comparing Glucocorticoid therapy and no Glucocorticoid therapy, CRRT and no CRRT are from χ² test, Fisher’s exact test, or Mann-Whitney U test. COVID-2019, coronavirus disease 2019; The severity was staged based on the guidelines for diagnosis and treatment of COVID-19 (trial seventh edition) published by Chinese National Health Commission in February 4, 2020.
