# Supplementary material for: Optimizing Management to Reduce the Mortality of COVID-19: Experience From a Designated Hospital for Severely and Critically Ill Patients in China
Source: Front Med (Lausanne). 2021 Mar 10;8:582764. doi: 10.3389/fmed.2021.582764 (PMC7987780; doi:10.3389/fmed.2021.582764)
Supplement: Supplementary file 2 [file Table_2.DOCX]

**Supplemental Table 2. Characteristics and Treatments in Severe+Critically ill COVID-19 patients.**

|  | **All patients** | **Anticoagulation therapy** | | **P Value** | |
| --- | --- | --- | --- | --- | --- |
|  |  | **Yes** | **No** | |  |
|  | **(N=431)** | **(N=160)** | **(N=271)** | |  |
| **Demographic characteristics** |  |  |  | |  |
| **Age- yr** | 65 [23-92] | 68 [26-92] | 62 [23-91] | | <0.001 |
| **Age≥ 65** | 218 (50.6) | 108 (67.5) | 110 (40.6) | | <0.001 |
| **Gender-Female** | 211 (49.0) | 70 (43.8) | 141 (52.0) | | 0.111 |
| **Personal history** |  |  |  | |  |
| **Smoking history** | 7 (1.6) | 3 (1.9) | 4 (1.5) | | 0.714 |
| **Current smoker** | 3 (0.7) | 2 (1.2) | 1 (0.4) | | 0.558 |
| **Former smoker** | 4 (0.9) | 1 (0.6) | 3 (1.1) | | 1 |
| **Coexisting disorder** |  |  |  | |  |
| **Cardiovascular disease** | 39 (9.0) | 22 (13.8) | 17 (6.3) | | 0.014 |
| **Hypertension** | 145 (33.6) | 64 (40.0) | 81 (29.9) | | 0.035 |
| **Diabetes** | 80 (18.6) | 37 (23.1) | 43 (15.9) | | 0.072 |
| **Cerebrovascular disease** | 17 (3.9) | 12 (7.5) | 5 (1.8) | | 0.008 |
| **Chronic pulmonary disease** | 39 (9.0) | 22 (13.8) | 17 (6.3) | | 0.014 |
| **Chronic kidney disease** | 13 (3.0) | 7 (4.4) | 6 (2.2) | | 0.247 |
| **Chronic liver disease** | 36 (8.4) | 15 (9.4) | 21 (7.7) | | 0.591 |
| **Malignancy** | 18 (4.2) | 5 (3.1) | 13 (4.8) | | 0.465 |
| **Signs and symptoms** |  |  |  | |  |
| **Fever** | 324 (75.2) | 124 (77.5) | 200 (73.8) | | 0.421 |
| **Cough** | 346 (80.3) | 133 (83.1) | 213 (78.6) | | 0.263 |
| **Expectoration** | 261 (60.6) | 102 (63.7) | 159 (58.7) | | 0.309 |
| **Shortness of breath** | 206 (47.8) | 86 (53.8) | 120 (44.3) | | 0.059 |
| **Pharyngalgia** | 44 (10.2) | 14 (8.8) | 30 (11.1) | | 0.512 |
| **Rhinorrhoea** | 27 (6.3) | 8 (5.0) | 19 (7.0) | | 0.538 |
| **Fatigue** | 106 (24.6) | 52 (32.5) | 54 (19.9) | | 0.004 |
| **Chest pain** | 36 (8.4) | 12 (7.5) | 24 (8.9) | | 0.72 |
| **Diarrhea** | 94 (21.8) | 30 (18.8) | 64 (23.6) | | 0.277 |
| **Abdominal pain** | 14 (3.2) | 6 (3.8) | 8 (3.0) | | 0.78 |
| **Anorexia** | 93 (21.6) | 39 (24.4) | 54 (19.9) | | 0.279 |
| **Nausea or Vomiting** | 48 (11.1) | 19 (11.9) | 29 (10.7) | | 0.752 |
| **Myalgia** | 57 (13.2) | 27 (16.9) | 30 (11.1) | | 0.105 |
| **Headache** | 45 (10.4) | 20 (12.5) | 25 (9.2) | | 0.328 |
| **Respiratory rate, breaths per minute** | 22.00 [20.00, 30.00] | 21.00 [20.00, 29.25] | 22.00 [20.00, 30.00] | | 0.644 |
| **Pulse, beat per minute** | 84.00 [77.00, 95.00] | 87.00 [78.00, 96.00] | 82.00 [76.00, 95.00] | | 0.013 |
| **Median arterial pressure, mmHg** | 97.00 [89.33, 105.67] | 98.50 [89.58, 107.50] | 96.33 [89.17, 104.67] | | 0.175 |
| **percutaneous oxygen saturation, %** | 96.00 [92.00, 98.00] | 95.00 [91.00, 98.00] | 96.00 [93.00, 98.00] | | 0.008 |
| **Comorbidities** |  |  |  | |  |
| **Acute respiratory distress syndrome** | 99 (23.0) | 73 (45.6) | 26 (9.6) | | <0.001 |
| **Acute kidney injury** | 33 (7.7) | 29 (18.1) | 4 (1.5) | | <0.001 |
| **Acute heart failure** | 88 (21.9) | 73 (47.7) | 15 (6.0) | | <0.001 |
| **Sepsis** | 76 (17.6) | 54 (33.8) | 22 (8.1) | | <0.001 |
| **Hyper-glycaemia, %** | 219 (51.0) | 62 (38.8) | 157 (58.4) | | <0.001 |
| **Secondary Infection** | 16 (3.7) | 14 (8.8) | 2 (0.7) | | <0.001 |
| **Treatments** |  |  |  | |  |
| **Extracorporeal membrane oxygenation** | 4 (0.9) | 4 (2.5) | 0 (0.0) | | 0.019 |
| **Renal replacement therapy** | 31 (7.2) | 29 (18.1) | 2 (0.7) | | <0.001 |
| **Antiviral agents** | 397 (92.1) | 142 (88.8) | 255 (94.1) | | 0.063 |
| **Antibacterial agents** | 335 (77.7) | 144 (90.0) | 191 (70.5) | | <0.001 |
| **Glucocorticoids** | 238 (55.2) | 112 (70.0) | 126 (46.5) | | <0.001 |
| **Immunoglobulin** | 123 (28.5) | 74 (46.2) | 49 (18.1) | | <0.001 |
| **Hematologic tests** |  |  |  | |  |
| **Leukocyte count, ×10^9^/L** | 6.05 [4.79, 8.14] | 7.45 [5.76, 9.63] | 5.59 [4.45, 7.12] | | <0.001 |
| **Neutrophil count, ×10^9^/L** | 4.20 [2.83, 6.15] | 5.78 [3.82, 8.24] | 3.62 [2.63, 5.29] | | <0.001 |
| **Lymphocyte count, ×10^9^/L** | 1.07 [0.71, 1.47] | 0.85 [0.56, 1.21] | 1.21 [0.85, 1.58] | | <0.001 |
| **Platelet count, ×10^9^/L** | 230.00 [170.50, 301.00] | 203.00 [151.75, 291.75] | 242.00 [179.00, 309.00] | | 0.002 |
| **Hemoglobin, g/L** | 126.00 [115.00, 137.00] | 125.50 [109.50, 139.00] | 126.00 [117.00, 135.00] | | 0.294 |
| **Coagulation function** |  |  |  | |  |
| **Prothrombin time, s** | 13.80 [13.20, 14.50] | 14.10 [13.50, 15.22] | 13.60 [13.10, 14.17] | | <0.001 |
| **Activated partial thromboplastin time, s** | 38.15 [35.50, 41.27] | 38.60 [35.27, 42.60] | 37.90 [35.52, 40.48] | | 0.184 |
| **D-dimer, ug/ml FEU** | 0.97 [0.41, 2.62] | 2.33 [0.98, 7.80] | 0.62 [0.29, 1.44] | | <0.001 |
| **Fibrinogen, g/L** | 4.71 [3.66, 5.96] | 5.16 [3.96, 6.31] | 4.46 [3.46, 5.67] | | 0.001 |
| **Prothrombin activity, %** | 90.00 [81.00, 98.00] | 86.00 [74.75, 93.25] | 92.00 [85.25, 99.00] | | <0.001 |
| **Biochemical liver function** |  |  |  | |  |
| **Alanine aminotransferase, U/L** | 23.00 [14.50, 38.00] | 23.00 [16.00, 40.00] | 22.00 [14.00, 37.00] | | 0.268 |
| **Aspartate aminotransferase, U/L** | 26.00 [19.00, 39.00] | 30.00 [21.00, 44.25] | 23.00 [18.00, 35.50] | | 0.002 |
| **Total bilirubin, umol/L** | 8.70 [6.55, 12.95] | 10.80 [7.68, 14.62] | 7.90 [6.20, 11.10] | | <0.001 |
| **Albumin, g/L** | 34.30 [30.75, 38.60] | 32.45 [28.87, 35.80] | 36.00 [32.05, 40.60] | | <0.001 |
| **Pre-albumin, mg/L** | 198.00 [123.25, 258.25] | 143.00 [87.00, 228.00] | 230.00 [171.50, 274.00] | | <0.001 |
| **lactose dehydrogenase, U/L** | 279.00 [211.00, 384.00] | 341.00 [253.00, 460.75] | 246.00 [187.50, 318.50] | | <0.001 |
| **Biochemical renal function** |  |  |  | |  |
| **Creatinine, umol/L** | 69.00 [56.00, 84.00] | 75.50 [61.00, 99.00] | 66.00 [54.00, 79.00] | | <0.001 |
| **Blood urea nitrogen, mmol/L** | 4.60 [3.50, 6.35] | 5.70 [4.10, 8.33] | 4.30 [3.35, 5.30] | | <0.001 |
| **eGFR, ml/min/1.73m^2^** | 90.40 [75.55, 99.30] | 82.95 [61.38, 94.23] | 93.20 [85.95, 102.05] | | <0.001 |
| **Sodium, mmol/L** | 139.30 [136.20, 141.40] | 137.70 [134.75, 140.33] | 140.00 [137.75, 141.80] | | <0.001 |
| **Potassium, mmol/L** | 4.07 [3.69, 4.42] | 4.05 [3.63, 4.52] | 4.08 [3.70, 4.36] | | 0.699 |
| **Calcium, mmol/L** | 2.09 [2.01, 2.18] | 2.05 [1.98, 2.14] | 2.11 [2.04, 2.19] | | <0.001 |
| **Biochemical cardiac function** |  |  |  | |  |
| **Creatinine kinase, U/L** | 57.00 [36.00, 94.50] | 61.50 [32.75, 119.25] | 53.50 [38.00, 84.75] | | 0.556 |
| **high-sensitivity cardiac troponin I (hs-cTnI), pg/ml** | 5.70 [2.60, 14.10] | 13.80 [4.50, 32.90] | 3.90 [1.00, 8.50] | | <0.001 |
| **N-terminal pro-brain natriuretic peptide**  **(NT-****proBNP), pg/ml** | 145.00 [59.00, 451.00] | 364.00 [131.00, 1330.00] | 100.50 [44.75, 210.00] | | <0.001 |
| **Infection related indices** |  |  |  | |  |
| **hs-CRP, mg/L** | 21.15 [2.92, 71.57] | 56.35 [14.65, 105.62] | 8.75 [1.63, 48.77] | | <0.001 |
| **ESR, mm/h** | 28.00 [13.00, 47.75] | 35.50 [22.00, 58.25] | 19.00 [9.00, 40.00] | | <0.001 |
| **Serum ferritin, ug/L** | 638.20 [326.60, 1047.20] | 877.95 [500.48, 1714.33] | 446.20 [213.90, 660.30] | | <0.001 |
| **IL-6, pg/ml** | 5.79 [2.43, 20.10] | 17.40 [5.24, 38.23] | 3.70 [1.65, 8.59] | | <0.001 |
| **IL-1β, pg/ml** | 4.90 [4.90, 4.90] | 4.90 [4.90, 5.97] | 4.90 [4.90, 4.90] | | 0.002 |
| **IL2R, U/ml** | 589.00 [397.00, 917.00] | 725.00 [511.75, 1134.00] | 508.00 [348.00, 745.50] | | <0.001 |
| **IL-8, pg/ml** | 12.60 [7.50, 22.50] | 16.95 [10.50, 30.27] | 10.40 [6.40, 18.25] | | <0.001 |
| **IL-10, pg/ml** | 4.90 [4.90, 5.10] | 4.90 [4.90, 7.88] | 4.90 [4.90, 4.90] | | <0.001 |
| **TNF-α, pg/ml** | 8.80 [6.30, 11.50] | 10.35 [7.82, 14.17] | 7.70 [6.00, 10.10] | | <0.001 |
| **Procalcitonin, ng/ml** | 0.08 [0.06, 0.17] | 0.14 [0.08, 0.30] | 0.07 [0.05, 0.09] | | <0.001 |

Data are median (IQR), numbers (percentages) of patients. p values comparing Anticoagulation therapy and no Anticoagulation are from χ² test, Fisher’s exact test, or Mann-Whitney U test. COVID-2019, coronavirus disease 2019; The severity was staged based on the guidelines for diagnosis and treatment of COVID-19 (trial seventh edition) published by Chinese National Health Commission in February 4, 2020.
