# Supplementary material for: Optimizing Management to Reduce the Mortality of COVID-19: Experience From a Designated Hospital for Severely and Critically Ill Patients in China
Source: Front Med (Lausanne). 2021 Mar 10;8:582764. doi: 10.3389/fmed.2021.582764 (PMC7987780; doi:10.3389/fmed.2021.582764)
Supplement: Supplementary file 3 [file Table_3.DOCX]

**Supplemental Table 3. Characteristics and Treatments in all intubation patients.**

|  | **All patients** | **Glucocorticoid therapy** | | **P Value** | | **CRRT** | | **P Value** |
| --- | --- | --- | --- | --- | --- | --- | --- | --- |
|  |  | **Yes** | **No** | |  | **Yes** | **No** |  |
|  | **(N=61)** | **(N=47)** | **(N=14)** | |  | **(N=33)** | **(N=28)** |  |
| **Demographic characteristics** |  |  |  | |  |  |  |  |
| **Age- yr** | 70 [44-87] | 68 [44-87] | 74[47-86] | | 0.145 | 70 [44-86] | 69.5 [47-87] | 0.524 |
| **Age≥ 65** | 43 (70.5) | 31 (66.0) | 12 (85.7) | | 0.197 | 23 (69.7) | 20 (71.4) | 1 |
| **Gender-Female** | 17 (27.9) | 14 (29.8) | 3 (21.4) | | 0.738 | 9 (27.3) | 8 (28.6) | 1 |
| **Personal history** |  |  |  | |  |  |  |  |
| **Smoking history** | 3 (4.9) | 3 (6.4) | 0 (0.0) | | 1 | 2 (6.1) | 1 (3.6) | 1 |
| **Current smoker** | 2 (3.3) | 2 (4.3) | 0 (0.0) | | 1 | 2 (6.1) | 0 (0.0) | 0.495 |
| **Former smoker** | 1 (1.6) | 1 (2.1) | 0 (0.0) | | 1 | 0 (0.0) | 1 (3.6) | 0.459 |
| **Coexisting disorder** |  |  |  | |  |  |  |  |
| **Cardiovascular disease** | 7 (11.5) | 6 (12.8) | 1 (7.1) | | 1 | 3 (9.1) | 4 (14.3) | 0.693 |
| **Hypertension** | 28 (45.9) | 20 (42.6) | 8 (57.1) | | 0.375 | 17 (51.5) | 11 (39.3) | 0.441 |
| **Diabetes** | 12 (19.7) | 11 (23.4) | 1 (7.1) | | 0.264 | 8 (24.2) | 4 (14.3) | 0.519 |
| **Cerebrovascular disease** | 5 (8.2) | 4 (8.5) | 1 (7.1) | | 1 | 0 (0.0) | 5 (17.9) | 0.017 |
| **Chronic pulmonary disease** | 10 (16.4) | 6 (12.8) | 4 (28.6) | | 0.217 | 6 (18.2) | 4 (14.3) | 0.741 |
| **Chronic kidney disease** | 2 (3.3) | 2 (4.3) | 0 (0.0) | | 1 | 2 (6.1) | 0 (0.0) | 0.495 |
| **Chronic liver disease** | 4 (6.6) | 2 (4.3) | 2 (14.3) | | 0.223 | 4 (12.1) | 0 (0.0) | 0.118 |
| **Malignancy** | 2 (3.3) | 1 (2.1) | 1 (7.1) | | 0.409 | 1 (3.0) | 1 (3.6) | 1 |
| **Signs and symptoms** |  |  |  | |  |  |  |  |
| **Fever** | 51 (83.6) | 41 (87.2) | 10 (71.4) | | 0.217 | 28 (84.8) | 23 (82.1) | 1 |
| **Cough** | 46 (75.4) | 33 (70.2) | 13 (92.9) | | 0.155 | 26 (78.8) | 20 (71.4) | 0.561 |
| **Expectoration** | 37 (60.7) | 25 (53.2) | 12 (85.7) | | 0.033 | 23 (69.7) | 14 (50.0) | 0.188 |
| **Shortness of breath** | 36 (59.0) | 23 (48.9) | 13 (92.9) | | 0.004 | 21 (63.6) | 15 (53.6) | 0.447 |
| **Pharyngalgia** | 6 (9.8) | 6 (12.8) | 0 (0.0) | | 0.321 | 4 (12.1) | 2 (7.1) | 0.678 |
| **Rhinorrhoea** | 1 (1.6) | 1 (2.1) | 0 (0.0) | | 1 | 0 (0.0) | 1 (3.6) | 0.459 |
| **Fatigue** | 23 (37.7) | 16 (34.0) | 7 (50.0) | | 0.351 | 13 (39.4) | 10 (35.7) | 0.797 |
| **Chest pain** | 5 (8.2) | 4 (8.5) | 1 (7.1) | | 1 | 3 (9.1) | 2 (7.1) | 1 |
| **Diarrhea** | 13 (21.3) | 9 (19.1) | 4 (28.6) | | 0.472 | 5 (15.2) | 8 (28.6) | 0.227 |
| **Abdominal pain** | 3 (4.9) | 2 (4.3) | 1 (7.1) | | 0.549 | 1 (3.0) | 2 (7.1) | 0.589 |
| **Anorexia** | 17 (27.9) | 13 (27.7) | 4 (28.6) | | 1 | 9 (27.3) | 8 (28.6) | 1 |
| **Nausea or Vomiting** | 4 (6.6) | 4 (8.5) | 0 (0.0) | | 0.565 | 2 (6.1) | 2 (7.1) | 1 |
| **Myalgia** | 11 (18.0) | 9 (19.1) | 2 (14.3) | | 1 | 7 (21.2) | 4 (14.3) | 0.526 |
| **Headache** | 10 (16.4) | 10 (21.3) | 0 (0.0) | | 0.098 | 6 (18.2) | 4 (14.3) | 0.741 |
| **Respiratory rate, breaths per minute** | 21.00 [20.00, 26.00] | 22.00 [20.00, 29.50] | 20.00 [20.00, 22.50] | | 0.155 | 22.00 [20.00, 30.00] | 20.50 [20.00, 25.25] | 0.906 |
| **Pulse, beat per minute** | 88.00 [78.00, 97.00] | 91.00 [82.00, 99.00] | 79.00 [77.25, 90.00] | | 0.022 | 90.00 [79.00, 99.00] | 88.00 [78.00, 96.25] | 0.75 |
| **Median arterial pressure, mmHg** | 96.33 [87.33, 106.33] | 95.33 [87.50, 104.50] | 99.00 [86.25, 109.42] | | 0.44 | 94.00 [86.67, 101.33] | 100.00 [91.83, 108.92] | 0.057 |
| **percutaneous oxygen saturation, %** | 94.00 [89.00, 98.00] | 92.00 [88.50, 98.00] | 94.50 [91.25, 98.00] | | 0.414 | 95.00 [90.00, 98.00] | 92.00 [88.75, 99.25] | 0.896 |
| **Comorbidities** |  |  |  | |  |  |  |  |
| **Acute respiratory distress syndrome** | 61 (100.0) | 47 (100.0) | 14 (100.0) | | 1 | 33 (100.0) | 28 (100.0) | 1 |
| **Acute kidney injury** | 31 (50.8) | 21 (44.7) | 10 (71.4) | | 0.127 | 20 (60.6) | 11 (39.3) | 0.126 |
| **Acute heart failure** | 54 (88.5) | 40 (85.1) | 14 (100.0) | | 0.187 | 30 (90.9) | 24 (85.7) | 0.693 |
| **Sepsis** | 55 (90.2) | 42 (89.4) | 13 (92.9) | | 1 | 29 (87.9) | 26 (92.9) | 0.678 |
| **Hyper-glycaemia, %** | 21 (34.4) | 15 (31.9) | 6 (42.9) | | 0.527 | 8 (24.2) | 13 (46.4) | 0.105 |
| **Secondary Infection** | 16 (26.2) | 13 (27.3) | 3 (21.4) | | 0.742 | 9 (27.3) | 7 (25.0) | 1 |
| **Treatments** |  |  |  | |  |  |  |  |
| **Extracorporeal membrane oxygenation** | 6 (9.8) | 6 (12.8) | 0 (0.0) | | 0.321 | 6 (18.2) | 0 (0.0) | 0.027 |
| **Renal replacement therapy** | 33 (54.1) | 27 (57.4) | 6 (42.9) | | 0.375 | 33 (100.0) | 0 (0.0) | <0.001 |
| **Antiviral agents** | 51 (83.6) | 40 (85.1) | 11 (78.6) | | 0.683 | 29 (87.9) | 22 (78.6) | 0.49 |
| **Antibacterial agents** | 59 (96.7) | 46 (97.9) | 13 (92.9) | | 0.409 | 33 (100.0) | 26 (92.9) | 0.207 |
| **Glucocorticoids** | 47 (77.0) | 47 (100.0) | 0 (0.0) | | <0.001 | 27 (81.8) | 20 (71.4) | 0.375 |
| **Immunoglobulin** | 51 (83.6) | 40 (85.1) | 11 (78.6) | | 0.683 | 31 (93.9) | 20 (71.4) | 0.034 |
| **Hematologic tests** |  |  |  | |  |  |  |  |
| **Leukocyte count, ×10^9^/L** | 7.95 [5.70, 10.13] | 7.95 [5.50, 9.60] | 7.96 [6.09, 13.55] | | 0.377 | 7.51 [4.77, 9.48] | 8.07 [6.64, 10.38] | 0.321 |
| **Neutrophil count, ×10^9^/L** | 6.50 [4.03, 8.86] | 6.50 [3.84, 8.68] | 6.89 [4.83, 11.79] | | 0.391 | 6.07 [3.14, 8.53] | 6.83 [5.02, 9.19] | 0.291 |
| **Lymphocyte count, ×10^9^/L** | 0.71 [0.49, 0.95] | 0.61 [0.48, 0.83] | 0.88 [0.74, 1.00] | | 0.118 | 0.71 [0.48, 0.98] | 0.72 [0.49, 0.90] | 0.96 |
| **Platelet count, ×10^9^/L** | 163.00 [110.00, 235.00] | 182.00 [121.00, 265.00] | 147.00 [86.00, 169.50] | | 0.131 | 152.00 [117.00, 200.00] | 195.00 [108.50, 293.50] | 0.259 |
| **Hemoglobin, g/L** | 138.00 [119.00, 145.00] | 139.00 [121.00, 146.00] | 131.00 [117.25, 138.75] | | 0.236 | 138.00 [125.00, 144.00] | 137.00 [116.75, 145.50] | 0.937 |
| **Coagulation function** |  |  |  | |  |  |  |  |
| **Prothrombin time, s** | 14.80 [13.80, 16.10] | 14.80 [13.75, 15.75] | 15.20 [14.00, 16.67] | | 0.315 | 15.00 [13.80, 16.20] | 14.70 [13.85, 15.88] | 0.931 |
| **Activated partial thromboplastin time, s** | 39.70 [35.90, 43.70] | 41.50 [36.15, 44.15] | 36.60 [34.52, 41.25] | | 0.15 | 39.90 [36.10, 43.40] | 39.50 [35.60, 43.93] | 0.988 |
| **D-dimer, ug/ml FEU** | 2.80 [1.30, 18.06] | 2.41 [1.12, 10.30] | 9.02 [4.38, 22.00] | | 0.027 | 2.71 [0.98, 18.06] | 3.88 [1.72, 15.42] | 0.432 |
| **Fibrinogen, g/L** | 5.18 [4.12, 6.22] | 5.20 [4.54, 6.34] | 3.80 [2.33, 5.42] | | 0.062 | 5.08 [3.99, 5.75] | 5.29 [4.48, 7.00] | 0.281 |
| **Prothrombin activity, %** | 78.00 [67.00, 90.00] | 78.00 [70.00, 90.00] | 74.50 [64.00, 87.00] | | 0.299 | 77.00 [67.00, 90.00] | 79.00 [68.50, 88.50] | 0.937 |
| **Biochemical liver function** |  |  |  | |  |  |  |  |
| **Alanine aminotransferase, U/L** | 28.00 [18.00, 46.00] | 28.00 [18.00, 42.50] | 32.50 [16.75, 54.50] | | 0.797 | 28.00 [19.00, 48.00] | 27.50 [16.00, 41.50] | 0.602 |
| **Aspartate aminotransferase, U/L** | 38.00 [26.00, 58.00] | 35.00 [25.00, 57.50] | 47.50 [33.75, 67.75] | | 0.217 | 39.00 [31.00, 58.00] | 34.50 [25.00, 56.75] | 0.612 |
| **Total bilirubin, umol/L** | 11.80 [9.10, 18.80] | 11.50 [8.95, 14.50] | 17.15 [9.95, 20.93] | | 0.207 | 11.80 [8.30, 16.50] | 12.00 [10.55, 18.98] | 0.271 |
| **Albumin, g/L** | 31.80 [29.50, 33.90] | 31.70 [29.70, 33.45] | 32.85 [29.45, 36.05] | | 0.445 | 31.80 [29.50, 33.50] | 31.70 [30.07, 34.70] | 0.811 |
| **Pre-albumin, mg/L** | 91.50 [79.00, 138.50] | 90.00 [79.00, 146.00] | 101.00 [79.00, 137.00] | | 0.701 | 90.00 [79.00, 134.00] | 93.00 [79.00, 138.00] | 0.936 |
| **lactose dehydrogenase, U/L** | 441.00 [304.00, 588.00] | 431.00 [305.50, 553.50] | 561.00 [275.25, 817.25] | | 0.295 | 435.00 [304.00, 586.00] | 452.00 [304.25, 694.75] | 0.712 |
| **Biochemical renal function** |  |  |  | |  |  |  |  |
| **Creatinine, umol/L** | 82.00 [66.00, 108.00] | 79.00 [61.50, 108.50] | 83.50 [76.75, 105.50] | | 0.345 | 88.00 [66.00, 117.00] | 79.00 [63.50, 101.25] | 0.271 |
| **Blood urea nitrogen, mmol/L** | 7.50 [5.10, 10.40] | 7.50 [4.95, 10.35] | 7.45 [5.57, 11.48] | | 0.619 | 7.80 [5.00, 10.30] | 7.10 [5.25, 10.45] | 0.994 |
| **eGFR, ml/min/1.73m^2^** | 73.80 [54.80, 89.65] | 77.15 [57.83, 93.20] | 66.75 [51.10, 78.67] | | 0.255 | 74.90 [51.35, 86.55] | 72.70 [61.70, 95.90] | 0.36 |
| **Sodium, mmol/L** | 137.90 [134.10, 140.30] | 137.10 [133.15, 139.65] | 139.65 [137.35, 142.28] | | 0.033 | 136.50 [133.50, 139.90] | 139.00 [137.00, 140.93] | 0.068 |
| **Potassium, mmol/L** | 4.18 [3.68, 4.72] | 4.18 [3.68, 4.69] | 4.23 [3.65, 4.82] | | 0.504 | 4.14 [3.45, 4.71] | 4.23 [3.74, 4.74] | 0.347 |
| **Calcium, mmol/L** | 2.03 [1.98, 2.11] | 2.03 [1.98, 2.10] | 2.04 [1.99, 2.17] | | 0.345 | 2.01 [1.97, 2.06] | 2.07 [2.01, 2.20] | 0.012 |
| **Biochemical cardiac function** |  |  |  | |  |  |  |  |
| **Creatinine kinase, U/L** | 103.00 [48.00, 185.00] | 112.00 [46.00, 189.25] | 88.00 [50.00, 154.00] | | 0.977 | 77.50 [39.25, 272.50] | 106.00 [70.00, 151.00] | 0.643 |
| **high-sensitivity cardiac troponin I (hs-cTnI), pg/ml** | 19.20 [8.80, 87.00] | 14.60 [7.65, 63.40] | 31.55 [22.88, 344.80] | | 0.014 | 18.60 [8.00, 75.40] | 24.65 [10.30, 118.65] | 0.524 |
| **N-terminal pro-brain natriuretic peptide**  **(NT-****proBNP), pg/ml** | 800.00 [303.00, 1496.00] | 606.00 [208.00, 1109.00] | 1386.00 [1048.75, 2742.00] | | 0.005 | 523.00 [230.00, 1099.00] | 964.50 [621.00, 2029.00] | 0.056 |
| **Infection related indices** |  |  |  | |  |  |  |  |
| **hs-CRP, mg/L** | 81.90 [46.10, 126.20] | 81.90 [40.65, 124.90] | 84.50 [52.67, 139.18] | | 0.817 | 70.40 [37.60, 115.70] | 97.25 [50.00, 150.35] | 0.275 |
| **ESR, mm/h** | 32.00 [16.00, 55.50] | 35.50 [21.25, 55.75] | 19.00 [9.00, 25.00] | | 0.147 | 30.00 [14.00, 54.00] | 36.00 [20.50, 70.25] | 0.39 |
| **Serum ferritin, ug/L** | 1298.10 [799.30, 2022.50] | 1298.10 [903.70, 2022.50] | 1090.35 [484.82, 1916.32] | | 0.361 | 1380.00 [960.00, 2162.40] | 1018.05 [666.98, 1864.67] | 0.3 |
| **IL-6, pg/ml** | 33.97 [16.59, 73.59] | 33.97 [17.44, 64.97] | 34.45 [14.89, 134.85] | | 0.91 | 33.97 [19.30, 62.30] | 34.47 [14.30, 137.55] | 0.76 |
| **IL-1β, pg/ml** | 4.90 [4.90, 6.50] | 4.90 [4.90, 6.50] | 4.90 [4.90, 6.53] | | 0.881 | 4.90 [4.90, 6.60] | 4.90 [4.90, 6.33] | 0.786 |
| **IL2R, U/ml** | 947.00 [634.00, 1275.50] | 940.00 [634.00, 1275.50] | 976.50 [760.25, 1183.75] | | 0.756 | 964.00 [717.00, 1412.00] | 840.50 [566.25, 1096.75] | 0.321 |
| **IL-8, pg/ml** | 21.90 [14.40, 38.20] | 21.90 [13.40, 38.20] | 21.80 [16.43, 39.55] | | 0.658 | 21.40 [12.50, 39.20] | 22.15 [16.27, 35.55] | 0.604 |
| **IL-10, pg/ml** | 6.60 [4.90, 10.35] | 7.00 [4.90, 11.85] | 4.90 [4.90, 8.90] | | 0.068 | 5.70 [4.90, 10.10] | 7.20 [5.23, 11.32] | 0.338 |
| **TNF-α, pg/ml** | 11.00 [7.95, 14.05] | 10.60 [7.90, 13.75] | 12.05 [10.22, 16.52] | | 0.224 | 11.00 [8.20, 13.80] | 10.90 [7.93, 14.38] | 0.915 |
| **Procalcitonin, ng/ml** | 0.18 [0.13, 0.36] | 0.18 [0.13, 0.35] | 0.17 [0.13, 0.40] | | 0.784 | 0.21 [0.13, 0.35] | 0.16 [0.14, 0.43] | 0.862 |

Data are median (IQR), numbers (percentages) of patients. p values comparing Glucocorticoid therapy and no Glucocorticoid therapy, CRRT and no CRRT are from χ² test, Fisher’s exact test, or Mann-Whitney U test. COVID-2019, coronavirus disease 2019; The severity was staged based on the guidelines for diagnosis and treatment of COVID-19 (trial seventh edition) published by Chinese National Health Commission in February 4, 2020.
