# Supplementary material for: Optimizing Management to Reduce the Mortality of COVID-19: Experience From a Designated Hospital for Severely and Critically Ill Patients in China
Source: Front Med (Lausanne). 2021 Mar 10;8:582764. doi: 10.3389/fmed.2021.582764 (PMC7987780; doi:10.3389/fmed.2021.582764)
Supplement: Supplementary file 4 [file Table_4.DOCX]

**Supplemental Table 4. Characteristics and Treatments in all intubation patients.**

|  | **All patients** | **Anticoagulation therapy** | | **P Value** | |
| --- | --- | --- | --- | --- | --- |
|  |  | **Yes** | **No** | |  |
|  | **(N=61)** | **(N=60)** | **(N=1)** | |  |
| **Demographic characteristics** |  |  |  | |  |
| **Age- yr** | 70 [44-87] | 70 [44-87] | 47[47-47] | | 0.099 |
| **Age≥ 65** | 43 (70.5) | 43 (71.7) | 0 (0.0) | | 0.295 |
| **Gender-Female** | 17 (27.9) | 17 (28.3) | 0 (0.0) | | 1 |
| **Personal history** |  |  |  | |  |
| **Smoking history** | 3 (4.9) | 3 (5.0) | 0 (0.0) | | 1 |
| **Current smoker** | 2 (3.3) | 2 (3.3) | 0 (0.0) | | 1 |
| **Former smoker** | 1 (1.6) | 1 (1.7) | 0 (0.0) | | 1 |
| **Coexisting disorder** |  |  |  | |  |
| **Cardiovascular disease** | 7 (11.5) | 7 (11.7) | 0 (0.0) | | 1 |
| **Hypertension** | 28 (45.9) | 27 (45.0) | 1 (100.0) | | 0.459 |
| **Diabetes** | 12 (19.7) | 12 (20.0) | 0 (0.0) | | 1 |
| **Cerebrovascular disease** | 5 (8.2) | 5 (8.3) | 0 (0.0) | | 1 |
| **Chronic pulmonary disease** | 10 (16.4) | 10 (16.7) | 0 (0.0) | | 1 |
| **Chronic kidney disease** | 2 (3.3) | 2 (3.3) | 0 (0.0) | | 1 |
| **Chronic liver disease** | 4 (6.6) | 4 (6.7) | 0 (0.0) | | 1 |
| **Malignancy** | 2 (3.3) | 2 (3.3) | 0 (0.0) | | 1 |
| **Signs and symptoms** |  |  |  | |  |
| **Fever** | 51 (83.6) | 51 (85.0) | 0 (0.0) | | 0.164 |
| **Cough** | 46 (75.4) | 45 (75.0) | 1 (100.0) | | 1 |
| **Expectoration** | 37 (60.7) | 37 (61.7) | 0 (0.0) | | 0.393 |
| **Shortness of breath** | 36 (59.0) | 35 (58.3) | 1 (100.0) | | 1 |
| **Pharyngalgia** | 6 (9.8) | 6 (10.0) | 0 (0.0) | | 1 |
| **Rhinorrhoea** | 1 (1.6) | 1 (1.7) | 0 (0.0) | | 1 |
| **Fatigue** | 23 (37.7) | 23 (38.3) | 0 (0.0) | | 1 |
| **Chest pain** | 5 (8.2) | 5 (8.3) | 0 (0.0) | | 1 |
| **Diarrhea** | 13 (21.3) | 12 (20.0) | 1 (100.0) | | 0.213 |
| **Abdominal pain** | 3 (4.9) | 3 (5.0) | 0 (0.0) | | 1 |
| **Anorexia** | 17 (27.9) | 17 (28.3) | 0 (0.0) | | 1 |
| **Nausea or Vomiting** | 4 (6.6) | 4 (6.7) | 0 (0.0) | | 1 |
| **Myalgia** | 11 (18.0) | 11 (18.3) | 0 (0.0) | | 1 |
| **Headache** | 10 (16.4) | 10 (16.7) | 0 (0.0) | | 1 |
| **Respiratory rate, breaths per minute** | 21.00 [20.00, 26.00] | 21.50 [20.00, 26.75] | 20.00 [20.00, 20.00] | | 0.525 |
| **Pulse, beat per minute** | 88.00 [78.00, 97.00] | 89.00 [78.75, 97.50] | 68.00 [68.00, 68.00] | | 0.139 |
| **Median arterial pressure, mmHg** | 96.33 [87.33, 106.33] | 96.50 [87.58, 106.50] | 84.33 [84.33, 84.33] | | 0.268 |
| **percutaneous oxygen saturation, %** | 94.00 [89.00, 98.00] | 94.50 [89.75, 98.00] | 88.00 [88.00, 88.00] | | 0.333 |
| **Comorbidities** |  |  |  | |  |
| **Acute respiratory distress syndrome** | 61 (100.0) | 60 (100.0) | 1 (100.0) | | 1 |
| **Acute kidney injury** | 31 (50.8) | 31 (51.7) | 0 (0.0) | | 0.492 |
| **Acute heart failure** | 54 (88.5) | 53 (88.3) | 1 (100.0) | | 1 |
| **Sepsis** | 55 (90.2) | 54 (90.0) | 1 (100.0) | | 1 |
| **Hyper-glycaemia, %** | 21 (34.4) | 20 (33.3) | 1 (100.0) | | 0.344 |
| **Secondary Infection** | 16 (26.2) | 16 (26.7) | 0 (0.0) | | 1 |
| **Treatments** |  |  |  | |  |
| **Extracorporeal membrane oxygenation** | 6 (9.8) | 6 (10.0) | 0 (0.0) | | 1 |
| **Renal replacement therapy** | 33 (54.1) | 33 (55.0) | 0 (0.0) | | 0.459 |
| **Antiviral agents** | 51 (83.6) | 50 (83.3) | 1 (100.0) | | 1 |
| **Antibacterial agents** | 59 (96.7) | 58 (96.7) | 1 (100.0) | | 1 |
| **Glucocorticoids** | 47 (77.0) | 47 (78.3) | 0 (0.0) | | 0.23 |
| **Immunoglobulin** | 51 (83.6) | 51 (85.0) | 0 (0.0) | | 0.164 |
| **Hematologic tests** |  |  |  | |  |
| **Leukocyte count, ×10^9^/L** | 7.95 [5.70, 10.13] | 7.98 [5.65, 10.14] | 7.27 [7.27, 7.27] | | 0.733 |
| **Neutrophil count, ×10^9^/L** | 6.50 [4.03, 8.86] | 6.55 [4.01, 8.93] | 5.82 [5.82, 5.82] | | 0.776 |
| **Lymphocyte count, ×10^9^/L** | 0.71 [0.49, 0.95] | 0.71 [0.49, 0.95] | 0.85 [0.85, 0.85] | | 0.532 |
| **Platelet count, ×10^9^/L** | 163.00 [110.00, 235.00] | 161.00 [109.50, 234.25] | 306.00 [306.00, 306.00] | | 0.201 |
| **Hemoglobin, g/L** | 138.00 [119.00, 145.00] | 138.00 [119.00, 145.00] | 133.00 [133.00, 133.00] | | 0.798 |
| **Coagulation function** |  |  |  | |  |
| **Prothrombin time, s** | 14.80 [13.80, 16.10] | 14.90 [13.78, 16.10] | 14.30 [14.30, 14.30] | | 0.733 |
| **Activated partial thromboplastin time, s** | 39.70 [35.90, 43.70] | 39.80 [36.05, 43.93] | 33.30 [33.30, 33.30] | | 0.191 |
| **D-dimer, ug/ml FEU** | 2.80 [1.30, 18.06] | 2.77 [1.29, 14.94] | 22.00 [22.00, 22.00] | | 0.179 |
| **Fibrinogen, g/L** | 5.18 [4.12, 6.22] | 5.13 [4.09, 6.28] | 5.39 [5.39, 5.39] | | 0.865 |
| **Prothrombin activity, %** | 78.00 [67.00, 90.00] | 77.50 [67.00, 90.00] | 84.00 [84.00, 84.00] | | 0.712 |
| **Biochemical liver function** |  |  |  | |  |
| **Alanine aminotransferase, U/L** | 28.00 [18.00, 46.00] | 27.50 [18.00, 45.25] | 47.00 [47.00, 47.00] | | 0.363 |
| **Aspartate aminotransferase, U/L** | 38.00 [26.00, 58.00] | 37.50 [25.75, 58.00] | 91.00 [91.00, 91.00] | | 0.155 |
| **Total bilirubin, umol/L** | 11.80 [9.10, 18.80] | 11.95 [9.07, 18.98] | 9.80 [9.80, 9.80] | | 0.443 |
| **Albumin, g/L** | 31.80 [29.50, 33.90] | 31.80 [29.40, 34.07] | 31.20 [31.20, 31.20] | | 0.691 |
| **Pre-albumin, mg/L** | 91.50 [79.00, 138.50] | 93.00 [79.00, 139.00] | 79.00 [79.00, 79.00] | | 0.269 |
| **lactose dehydrogenase, U/L** | 441.00 [304.00, 588.00] | 438.00 [302.50, 586.50] | 827.00 [827.00, 827.00] | | 0.173 |
| **Biochemical renal function** |  |  |  | |  |
| **Creatinine, umol/L** | 82.00 [66.00, 108.00] | 81.50 [65.75, 108.25] | 101.00 [101.00, 101.00] | | 0.589 |
| **Blood urea nitrogen, mmol/L** | 7.50 [5.10, 10.40] | 7.35 [5.07, 10.43] | 7.80 [7.80, 7.80] | | 0.887 |
| **eGFR, ml/min/1.73m^2^** | 73.80 [54.80, 89.65] | 72.70 [54.10, 89.80] | 76.00 [76.00, 76.00] | | 0.926 |
| **Sodium, mmol/L** | 137.90 [134.10, 140.30] | 137.80 [133.97, 140.07] | 142.30 [142.30, 142.30] | | 0.211 |
| **Potassium, mmol/L** | 4.18 [3.68, 4.72] | 4.17 [3.67, 4.71] | 5.00 [5.00, 5.00] | | 0.156 |
| **Calcium, mmol/L** | 2.03 [1.98, 2.11] | 2.04 [1.98, 2.11] | 2.03 [2.03, 2.03] | | 0.977 |
| **Biochemical cardiac function** |  |  |  | |  |
| **Creatinine kinase, U/L** | 103.00 [48.00, 185.00] | 99.50 [47.25, 181.25] | 1248.00 [1248.00, 1248.00] | | 0.101 |
| **high-sensitivity cardiac troponin I (hs-cTnI), pg/ml** | 19.20 [8.80, 87.00] | 20.30 [8.60, 94.53] | 11.80 [11.80, 11.80] | | 0.65 |
| **N-terminal pro-brain natriuretic peptide**  **(NT-****proBNP), pg/ml** | 800.00 [303.00, 1496.00] | 790.00 [302.75, 1448.00] | 1602.00 [1602.00, 1602.00] | | 0.334 |
| **Infection related indices** |  |  |  | |  |
| **hs-CRP, mg/L** | 81.90 [46.10, 126.20] | 85.80 [44.93, 130.05] | 58.00 [58.00, 58.00] | | 0.57 |
| **ESR, mm/h** | 32.00 [16.00, 55.50] | 32.00 [16.00, 55.50] | - | | - |
| **Serum ferritin, ug/L** | 1298.10 [799.30, 2022.50] | 1298.10 [799.30, 2022.50] | - | | - |
| **IL-6, pg/ml** | 33.97 [16.59, 73.59] | 33.97 [16.59, 73.59] | - | | - |
| **IL-1β, pg/ml** | 4.90 [4.90, 6.50] | 4.90 [4.90, 6.50] | - | | - |
| **IL2R, U/ml** | 947.00 [634.00, 1275.50] | 947.00 [634.00, 1275.50] | - | | - |
| **IL-8, pg/ml** | 21.90 [14.40, 38.20] | 21.90 [14.40, 38.20] | - | | - |
| **IL-10, pg/ml** | 6.60 [4.90, 10.35] | 6.60 [4.90, 10.35] | - | | - |
| **TNF-α, pg/ml** | 11.00 [7.95, 14.05] | 11.00 [7.95, 14.05] | - | | - |
| **Procalcitonin, ng/ml** | 0.18 [0.13, 0.36] | 0.17 [0.13, 0.37] | 0.18 [0.18, 0.18] | | 0.977 |

Data are median (IQR), numbers (percentages) of patients. p values comparing Anticoagulation therapy and no Anticoagulation therapy are from χ² test, Fisher’s exact test, or Mann-Whitney U test. COVID-2019, coronavirus disease 2019; The severity was staged based on the guidelines for diagnosis and treatment of COVID-19 (trial seventh edition) published by Chinese National Health Commission in February 4, 2020.
