# Supplementary material for: Optimizing Management to Reduce the Mortality of COVID-19: Experience From a Designated Hospital for Severely and Critically Ill Patients in China
Source: Front Med (Lausanne). 2021 Mar 10;8:582764. doi: 10.3389/fmed.2021.582764 (PMC7987780; doi:10.3389/fmed.2021.582764)
Supplement: Supplementary file 5 [file Table_5.DOCX]

Supplemental Table 5. Clinical Characteristics of six COVID-19-infected patients with ECMO treatment

| **Variables** | **Patient 1** | **Patient 2** | **Patient 3** | **Patient 4** | **Patient 5** | **Patient 6** |  |
| --- | --- | --- | --- | --- | --- | --- | --- |
| Age, years | 68 | 58 | 44 | 50 | 58 | 58 |  |
| Gender | Male | Male | Male | Male | Male | Female |  |
| History of chronic medical illness | Diabetes | Hypertension | Thyroid gland cancer | Diabetes  Chronic bronchitis | Hypertension  Diabetes | Hypertension  Diabetes |  |
| Duration of illness, days | 4 | 2 | 9 | 11 | 25 | 32 |  |
| Symptoms | Fever | Cough  Expectoration | Fever  Cough  Expectoration  Shortness of breath | Fever  Cough  Expectoration  Shortness of breath | Fever  Cough  Expectoration | Shortness of breath |  |
| **Vital signs on admission** | | | | | | | |
| T, °C | 36.5 | 36.5 | 39.2 | 36.4 | 36.5 | 36.5 |  |
| Respiratory rate, bpm | 20 | 18 | 18 | 20 | 38 | 34 |  |
| Pulse, bpm | 86 | 95 | 90 | 82 | 154 | 114 |  |
| Blood pressure, mmHg | 138/88 | 108/74 | 138/78 | 132/86 | 106/69 | N/A |  |
| SpO2, % | 96 | 98 | 95 | 98 | 79 (incubated) | 98 (mask 10 L/min) |  |
| **Biochemical liver function** | | | | | | |  |
| ALT, U/L | 19 | 32 | 145 | 54 | 72 | 45 |  |
| AST, U/L | 34 | 37 | 163 | 62 | 39 | 14 |  |
| Total bilirubin, umol/L | 6 | 5.8 | 9.1 | 8.6 | 58.5 | 7 |  |
| Direct bilirubin, umol/L | 3.4 | 3 | 3.9 | 4.7 | 46.1 | 3.1 |  |
| GGT, U/L | 29 | 51 | 95 | 78 | 126 | 81 |  |
| Albumin, g/L | 31.2 | 40.8 | 33.4 | 33.9 | 33.3 | 32.9 |  |
| Pre-albumin, mg/L |  |  |  |  |  |  |  |
| Total cholesterol, mmol/L | 2.68 | 4.91 | 3.84 | 3.19 | 3.25 | 4.74 |  |
| LDH, U/L | 307 | 242 | 315 | 588 | 446 | 366 |  |
| Cholinesterase, U/L |  |  |  |  |  |  |  |
| Glucose, mM | 5.98 | 7.92 | 7.03 | 6.89 | 10.7 | 9.23 |  |
| **Biochemical Renal function** | | | | | | |  |
| Creatinine, umol/L | 74 | 117 | 104 | 67 | 131 | 29 |  |
| Blood urea nitrogen, mmol/L | 3.9 | 8.2 | 5.1 | 2.9 | 11.9 | 3.2 |  |
| uric acid, umol/L | 205.9 | 480 | 190.7 | 177.4 | 195.7 | 68.2 |  |
| eGFR, ml/min/1.73m^2^ | 90.1 | 58.9 | 74.9 | 106.5 | 51.3 | 122.9 |  |
| Sodium, mmol/L | 131.8 | 133.6 | 132.9 | 139.9 | 140 | 136.9 |  |
| Potassium, mmol/L | 3.68 | 3.77 | 4.67 | 3.63 | 4.81 | 3.72 |  |
| Calcium, mmol/L | 1.92 | 2.09 | 2.04 | 2.1 | 2.04 | 2.06 |  |
| Chloride, mmol/L | 95.5 | 94.4 | 96 | 100.4 | 96.5 | 98.1 |  |
| **Coagulation function** | | | | | | |  |
| PT, s | 13.8 | 12.8 | 14 | 13.3 | 15.6 | 13.2 |  |
| APTT, s | 48.5 | 43.4 | 42.7 | 32.7 | 41.5 | 32.1 |  |
| D-dimer, ug/ml FEU | 0.39 | 0.32 | 0.6 | 1.94 | 22 | 3.33 |  |
| Fibrinogen, g/L | 5.03 | 3.99 | 5.08 | 5.7 | 5.4 | 5.18 |  |
| Prothrombin activity, % | 89 | 104 | 86 | 96 | 71 | 98 |  |
| INR | 1.07 | 0.97 | 1.09 | 1.02 | 1.25 | 1.01 |  |
| **Biochemical cardiac function** | | | | | | |  |
| Creatinine kinase, U/L | 20 | 417 | 74 | 67 | 57 | 35 |  |
| high-sensitivity cardiac troponin I (hs-cTnI), pg/ml | 18.6 | 9.2 | 2.5 | 17.1 | 296.4 | 3.8 |  |
| N-terminal pro-brain natriuretic peptide  (NT-proBNP), pg/ml | 800 | 69 | 37 | 2584 | 302 | 101 |  |
| Myoglobin, ng/ml | 35.2 | 228.1 | 43.5 | 67.6 | 156.9 | 66.5 |  |
| **Hematologic tests** | | | | | | |  |
| WBCs, ×10^9^/L | 3.68 | 4.56 | 3.25 | 7.07 | 30.41 | 12.12 |  |
| Hemoglobin, g/dL | 145 | 133 | 145 | 125 | 138 | 116 |  |
| Platelets, ×10^9^/L | 163 | 147 | 133 | 316 | 152 | 200 |  |
| Neutrophils, ×10^9^/L | 2.2 | 3.03 | 2.09 | 5.77 | 28.34 | 9.74 |  |
| Lymphocytes, ×10^9^/L | 0.88 | 1.19 | 0.71 | 0.81 | 0.59 | 1.37 |  |
| **Infection related indices** | | | | | | |  |
| hs-CRP, mg/L | 22.7 | 18.7 | 19.5 | 70.4 | 221 | 14.1 |  |
| ESR, mm/h | 30 | 21 | 56 | 54 | NA | 40 |  |
| Serum ferritin, ug/L | 1928 | 2360.9 | 1598.5 | 971.6 | 2322.9 | 1032.8 |  |
| IL-6, pg/ml | 52.92 | 25.43 | 16.98 | 10.52 | 81.04 | 28.71 |  |
| IL-1β, pg/ml | 7.1 | 4.9 | 12.7 | 4.9 | 6.9 | 4.9 |  |
| IL2R, U/ml | 1506 | 1091 | 401 | 388 | 1109 | 624 |  |
| IL-8, pg/ml | 27.4 | 9.4 | 4.9 | 4.9 | 24.6 | 39.2 |  |
| IL-10, pg/ml | 15.9 | 37.2 | 9.2 | 4.9 | 55.3 | 7.4 |  |
| TNF-α, pg/ml | 7.1 | 11.8 | 5.4 | 8.3 | 11.1 | 13.8 |  |
| Procalcitonin, ng/ml | 0.1 | 0.1 | 0.08 | 0.1 | 1.92 | 0.1 |  |
| **Initial parameters** | | | | | | |  |
| PaO_2_/FiO_2_, mmHg | 107 | N/A | 164 | 84 | 53 | 109 |  |
| Duration of ECMO | N/A | 14 | 6 | 9 | 16 | 7 |  |
| Clinical outcome | Transferred | Recovery | Recovery | Recovery | Death | Recovery |  |
