# Supplementary material for: Optimizing Management to Reduce the Mortality of COVID-19: Experience From a Designated Hospital for Severely and Critically Ill Patients in China
Source: Front Med (Lausanne). 2021 Mar 10;8:582764. doi: 10.3389/fmed.2021.582764 (PMC7987780; doi:10.3389/fmed.2021.582764)
Supplement: Supplementary file 6 [file Table_6.DOCX]

**Supplemental Table 6. Characteristics and Treatments in Severe and Critically ill COVID-19 patients.**

|  | **All patients** | **Severe** | | **Critically ill** | **P Value** |
| --- | --- | --- | --- | --- | --- |
|  | **(N=431)** | **(N=325)** | **(N=106)** | |  |
| **Demographic characteristics** |  |  |  | |  |
| **Age- yr** | 65 [23-92] | 64 [23-92] | 68 [32-91] | | <0.001 |
| **Age≥ 65** | 218 (50.6) | 150 (46.2) | 68 (64.2) | | 0.002 |
| **Gender-Female** | 211 (49.0) | 169 (52.0) | 42 (39.6) | | 0.033 |
| **Personal history** |  |  |  | |  |
| **Smoking history** | 7 (1.6) | 4 (1.2) | 3 (2.8) | | 0.37 |
| **Current smoker** | 3 (0.7) | 2 (0.6) | 1 (0.9) | | 0.572 |
| **Former smoker** | 4 (0.9) | 2 (0.6) | 2 (1.9) | | 0.254 |
| **Coexisting disorder** |  |  |  | |  |
| **Cardiovascular disease** | 39 (9.0) | 25 (7.7) | 14 (13.2) | | 0.117 |
| **Hypertension** | 145 (33.6) | 102 (31.4) | 43 (40.6) | | 0.097 |
| **Diabetes** | 80 (18.6) | 59 (18.2) | 21 (19.8) | | 0.774 |
| **Cerebrovascular disease** | 17 (3.9) | 8 (2.5) | 9 (8.5) | | 0.017 |
| **Chronic pulmonary disease** | 39 (9.0) | 17 (5.2) | 22 (20.8) | | <0.001 |
| **Chronic kidney disease** | 13 (3.0) | 8 (2.5) | 5 (4.7) | | 0.322 |
| **Malignancy** | 36 (8.4) | 29 (8.9) | 7 (6.6) | | 0.547 |
| **Chronic liver disease** | 18 (4.2) | 13 (4.0) | 5 (4.7) | | 0.781 |
| **Signs and symptoms** |  |  |  | |  |
| **Fever** | 324 (75.2) | 239 (73.5) | 85 (80.2) | | 0.196 |
| **Cough** | 346 (80.3) | 261 (80.3) | 85 (80.2) | | 1 |
| **Expectoration** | 261 (60.6) | 196 (60.3) | 65 (61.3) | | 0.909 |
| **Shortness of breath** | 206 (47.8) | 138 (42.5) | 68 (64.2) | | <0.001 |
| **Pharyngalgia** | 44 (10.2) | 35 (10.8) | 9 (8.5) | | 0.583 |
| **Rhinorrhoea** | 27 (6.3) | 24 (7.4) | 3 (2.8) | | 0.109 |
| **Fatigue** | 106 (24.6) | 77 (23.7) | 29 (27.4) | | 0.439 |
| **Chest pain** | 36 (8.4) | 27 (8.3) | 9 (8.5) | | 1 |
| **Diarrhea** | 94 (21.8) | 66 (20.3) | 28 (26.4) | | 0.222 |
| **Abdominal pain** | 14 (3.2) | 10 (3.1) | 4 (3.8) | | 0.754 |
| **Anorexia** | 93 (21.6) | 63 (19.4) | 30 (28.3) | | 0.058 |
| **Nausea or Vomiting** | 48 (11.1) | 36 (11.1) | 12 (11.3) | | 1 |
| **Myalgia** | 57 (13.2) | 42 (12.9) | 15 (14.2) | | 0.743 |
| **Headache** | 45 (10.4) | 31 (9.5) | 14 (13.2) | | 0.278 |
| **Respiratory rate, breaths per minute** | 22.00 [20.00, 30.00] | 22.00 [20.00, 30.00] | 21.00 [20.00, 26.00] | | 0.256 |
| **Pulse, beat per minute** | 84.00 [77.00, 95.00] | 84.00 [76.00, 94.00] | 86.00 [78.00, 98.00] | | 0.06 |
| **Median arterial pressure, mmHg** | 97.00 [89.33, 105.67] | 97.33 [90.00, 105.67] | 96.50 [86.67, 106.17] | | 0.177 |
| **percutaneous oxygen saturation, %** | 96.00 [92.00, 98.00] | 96.00 [93.00, 98.00] | 92.50 [88.00, 96.75] | | <0.001 |
| **Comorbidities** |  |  |  | |  |
| **Acute respiratory distress syndrome** | 242 (56.1) | 141 (43.4) | 101 (95.3) | | <0.001 |
| **Acute kidney injury** | 33 (7.7) | 6 (1.8) | 27 (25.5) | | <0.001 |
| **Acute heart failure** | 88 (21.9) | 0 (0.0) | 88 (84.6) | | <0.001 |
| **Sepsis** | 76 (17.6) | 19 (5.8) | 57 (53.8) | | <0.001 |
| **Secondary Infection** | 16 (3.7) | 2 (0.6) | 14 (13.2) | | <0.001 |
| **Treatments** |  |  |  | |  |
| **Extracorporeal membrane oxygenation** | 4 (0.9) | 1 (0.3) | 3 (2.8) | | 0.048 |
| **Renal replacement therapy** | 31 (7.2) | 4 (1.2) | 27 (25.5) | | <0.001 |
| **Antiviral agents** | 397 (92.1) | 306 (94.2) | 91 (85.8) | | 0.011 |
| **Antibacterial agents** | 335 (77.7) | 237 (72.9) | 98 (92.5) | | <0.001 |
| **Glucocorticoids** | 238 (55.2) | 157 (48.3) | 81 (76.4) | | <0.001 |
| **Immunoglobulin** | 123 (28.5) | 61 (18.8) | 62 (58.5) | | <0.001 |
| **Outcomes** |  |  |  | |  |
| **In-hospital death, %** | 45 (10.4) | 3 (0.9) | 42 (39.6) | | <0.001 |
| **Hematologic tests** |  |  |  | |  |
| **Leukocyte count, ×10^9^/L** | 6.05 [4.79, 8.14] | 5.77 [4.68, 7.46] | 7.98 [5.72, 10.60] | | <0.001 |
| **Neutrophil count, ×10^9^/L** | 4.20 [2.83, 6.15] | 3.76 [2.71, 5.48] | 6.26 [4.44, 9.38] | | <0.001 |
| **Lymphocyte count, ×10^9^/L** | 1.07 [0.71, 1.47] | 1.21 [0.89, 1.54] | 0.68 [0.48, 0.97] | | <0.001 |
| **Platelet count, ×10^9^/L** | 230.00 [170.50, 301.00] | 242.00 [180.00, 310.00] | 189.00 [133.75, 249.25] | | <0.001 |
| **Hemoglobin, g/L** | 126.00 [115.00, 137.00] | 126.00 [116.00, 135.00] | 125.50 [109.25, 140.75] | | 0.894 |
| **Coagulation function** |  |  |  | |  |
| **Prothrombin time, s** | 13.80 [13.20, 14.50] | 13.60 [13.10, 14.10] | 14.80 [13.80, 15.80] | | <0.001 |
| **Activated partial thromboplastin time, s** | 38.15 [35.50, 41.27] | 38.00 [35.40, 40.23] | 39.25 [35.68, 43.18] | | 0.01 |
| **D-dimer, ug/ml FEU** | 0.97 [0.41, 2.62] | 0.74 [0.32, 1.67] | 2.81 [1.30, 15.56] | | <0.001 |
| **Fibrinogen, g/L** | 4.71 [3.66, 5.96] | 4.60 [3.61, 5.96] | 5.05 [4.08, 5.97] | | 0.156 |
| **Prothrombin activity, %** | 90.00 [81.00, 98.00] | 92.00 [86.00, 99.00] | 78.00 [69.25, 90.00] | | <0.001 |
| **Biochemical liver function** |  |  |  | |  |
| **Alanine aminotransferase, U/L** | 23.00 [14.50, 38.00] | 21.00 [14.00, 37.00] | 27.50 [17.00, 40.75] | | 0.043 |
| **Aspartate aminotransferase, U/L** | 26.00 [19.00, 39.00] | 23.00 [18.00, 35.00] | 33.00 [24.00, 50.00] | | <0.001 |
| **Total bilirubin, umol/L** | 8.70 [6.55, 12.95] | 8.10 [6.20, 11.90] | 11.90 [8.30, 16.58] | | <0.001 |
| **Albumin, g/L** | 34.30 [30.75, 38.60] | 35.60 [32.20, 40.00] | 31.15 [28.20, 33.98] | | <0.001 |
| **Pre-albumin, mg/L** | 198.00 [123.25, 258.25] | 230.50 [182.75, 274.00] | 112.00 [79.00, 169.75] | | <0.001 |
| **lactose dehydrogenase, U/L** | 279.00 [211.00, 384.00] | 250.00 [191.00, 328.00] | 398.00 [277.50, 563.00] | | <0.001 |
| **Biochemical renal function** |  |  |  | |  |
| **Creatinine, umol/L** | 69.00 [56.00, 84.00] | 67.00 [55.00, 81.00] | 76.50 [62.50, 101.00] | | <0.001 |
| **Blood urea nitrogen, mmol/L** | 4.60 [3.50, 6.35] | 4.30 [3.30, 5.50] | 6.85 [4.32, 10.35] | | <0.001 |
| **eGFR, ml/min/1.73m^2^** | 90.40 [75.55, 99.30] | 91.85 [81.97, 100.62] | 82.90 [58.30, 94.25] | | <0.001 |
| **Sodium, mmol/L** | 139.30 [136.20, 141.40] | 139.50 [136.80, 141.50] | 138.25 [134.65, 140.93] | | 0.014 |
| **Potassium, mmol/L** | 4.07 [3.69, 4.42] | 4.07 [3.69, 4.37] | 4.06 [3.64, 4.61] | | 0.683 |
| **Calcium, mmol/L** | 2.09 [2.01, 2.18] | 2.12 [2.04, 2.19] | 2.02 [1.97, 2.09] | | <0.001 |
| **Biochemical cardiac function** |  |  |  | |  |
| **Creatinine kinase, U/L** | 57.00 [36.00, 94.50] | 53.00 [36.00, 77.00] | 73.00 [38.00, 160.00] | | 0.005 |
| **high-sensitivity cardiac troponin I (hs-cTnI), pg/ml** | 5.70 [2.60, 14.10] | 4.10 [2.00, 9.15] | 18.60 [7.50, 102.05] | | <0.001 |
| **N-terminal pro-brain natriuretic peptide**  **(NT-****proBNP), pg/ml** | 145.00 [59.00, 451.00] | 103.00 [48.00, 213.00] | 1073.00 [323.00, 2468.00] | | <0.001 |
| **Infection related indices** |  |  |  | |  |
| **hs-CRP, mg/L** | 21.15 [2.92, 71.57] | 11.05 [2.00, 52.95] | 68.10 [31.83, 119.58] | | <0.001 |
| **ESR, mm/h** | 28.00 [13.00, 47.75] | 22.00 [9.00, 44.00] | 36.00 [20.75, 54.25] | | 0.004 |
| **Serum ferritin, ug/L** | 638.20 [326.60, 1047.20] | 465.15 [258.17, 709.75] | 939.70 [675.10, 1844.00] | | <0.001 |
| **IL-6, pg/ml** | 5.79 [2.43, 20.10] | 3.88 [1.89, 10.54] | 21.27 [8.86, 52.77] | | <0.001 |
| **IL-1β, pg/ml** | 4.90 [4.90, 4.90] | 4.90 [4.90, 4.90] | 4.90 [4.90, 5.88] | | 0.047 |
| **IL2R, U/ml** | 589.00 [397.00, 917.00] | 545.00 [366.50, 792.00] | 829.50 [537.00, 1239.75] | | <0.001 |
| **IL-8, pg/ml** | 12.60 [7.50, 22.50] | 11.40 [6.85, 20.45] | 17.90 [10.77, 28.00] | | <0.001 |
| **IL-10, pg/ml** | 4.90 [4.90, 5.10] | 4.90 [4.90, 4.90] | 4.90 [4.90, 8.50] | | <0.001 |
| **TNF-α, pg/ml** | 8.80 [6.30, 11.50] | 8.30 [6.10, 10.65] | 10.65 [7.90, 14.38] | | <0.001 |
| **Procalcitonin, ng/ml** | 21.15 [2.92, 71.57] | 11.05 [2.00, 52.95] | 68.10 [31.83, 119.58] | | <0.001 |

Data are median (IQR), numbers (percentages) of patients. p values comparing severe and critically ill are from χ² test, Fisher’s exact test, or Mann-Whitney U test. COVID-2019, coronavirus disease 2019; The severity was staged based on the guidelines for diagnosis and treatment of COVID-19 (trial seventh edition) published by Chinese National Health Commission in February 4, 2020.
