# Supplementary figures and images for: Optimizing Management to Reduce the Mortality of COVID-19: Experience From a Designated Hospital for Severely and Critically Ill Patients in China
Source: Front Med (Lausanne). 2021 Mar 10;8:582764. doi: 10.3389/fmed.2021.582764 (PMC7987780; doi:10.3389/fmed.2021.582764)

ROC curves for SpO2/FiO2 and mortality

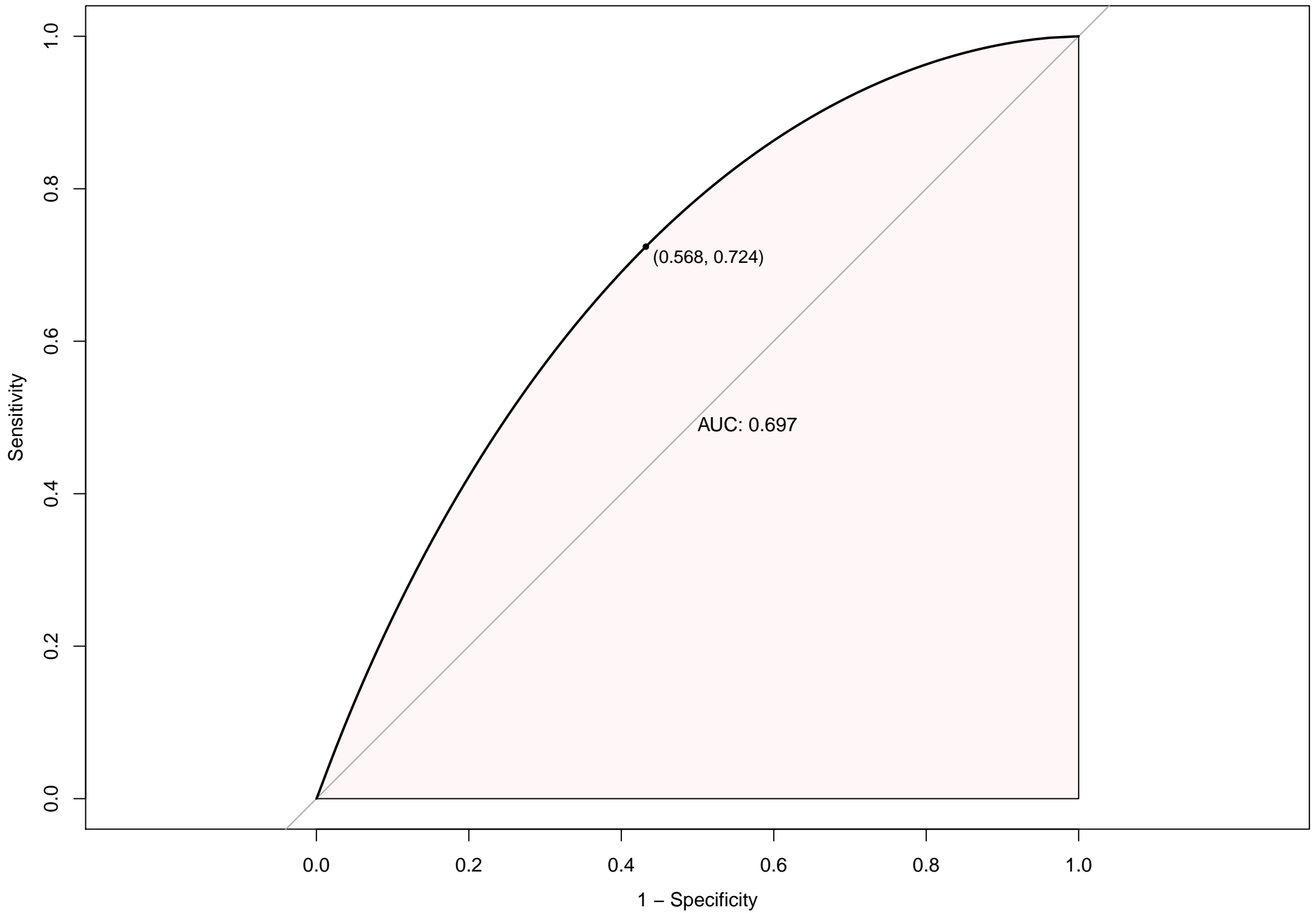

Supplement: Supplementary file 7 [file Data_Sheet_1.PDF]
