# Supplementary material for: Optimizing Management to Reduce the Mortality of COVID-19: Experience From a Designated Hospital for Severely and Critically Ill Patients in China
Source: Front Med (Lausanne). 2021 Mar 10;8:582764. doi: 10.3389/fmed.2021.582764 (PMC7987780; doi:10.3389/fmed.2021.582764)
Supplement: Supplementary file 8 [file Data_Sheet_2.PDF]

We cited our previous articles entitled “Organ function support in patients with coronavirus disease 2019: Tongji experience” published in Frontiers of Medicine:

Respiratory support:

For Non-Invasive Ventilation(NIV): critical type COVID-19 patients not only show acute respiratory distress syndrome (ARDS)-related ventilation/perfusion defects, but also manifest as diffuse dysfunction due to pulmonary interstitial disease and fibrosis. Therefore, oxygen saturation remains poor in some patients after tracheal intubation and invasive mechanical ventilation. Inability of weaning from mechanical ventilation also lead to complications such as ventilator-associated pneumonia in the later weeks of treatment. The mortality of patient with invasive mechanical ventilation remains high due to the above mentioned factors.

We recommend that patients with severe tendency who can tolerate NIV should be preferred for NIV assistance. The initial parameters could set to IPAP 8 - 10 cmH2O, EPAP 5 - 8 cmH2O, and FiO2 is 100%. The observation time of using a NIV is generally 2 hours [dynamic adjustment parameters during the period according to the patient's condition, tidal volume (Vt) and blood oxygen saturation. If this situation of Vt $\leq$ 9 ml / kg, RR $\leq$ 30 times / minute, PaO2 / FiO2 can be

maintained , NIV may continue ; if Vt 9 - 12 ml / kg, PaO_2 / FiO_2 is stable, you can closely monitor for 6 h, if Vt > 12 ml / kg, or PaO_2 / FiO_2 gets worse, you should immediately stop NIV and switch to tracheal intubation for invasive mechanical ventilation.

Invasive mechanical ventilation:

It should be converted to invasive mechanical ventilation in time when NIV is ineffective.

(1) finger pulse oxygen saturation ≤ 90% and or RR ≥ 30 times / min;

(2) hypercapnia, respiratory acidosis (pH ≤ 7.25);

(3) hemodynamic instability;

(4) multiple organ failure;

(5) disorders of consciousness;

(6) patients are extremely uncooperative.

Lung protective ventilation strategy should be adopted. It is recommended to use small tidal volume (4 - 8 ml /kg, ideal kg body weight) and low plateau pressure ventilation (< 30 cmH_2O).The initial tidal volume (Vt) can be set to 6 ml/kg. If ventilation is insufficient, the tidal volume can be appropriately increased to 8 ml/kg. After setting Vt, it is necessary to monitor the pressure index and control the pressure of the inhalation platform below 30 cmH_2O. When the airway platform pressure is ≤ 35 cmH_2O, high PEEP can be used appropriately. Airway warming and humidification should be emphasized to avoid blood and sputum scab

to block airways. If the plauteau pressure is > 30 cmH2O or the driving pressure is > 15 cmH2O, Vt should be gradually reduced at a speed of 1ml/kg until the suction plauteau pressure < 30cmH2O and the driving pressure < 15 cmH2O or Vt reduced to 4 ml/ kg (ideal weight) . At the same time lowering Vt and increasing RR to ensure alveolar minute ventilation and avoid CO2 retention. If the spontaneous breathing is too strong and the tidal volume is too large (> 8 ml / kg), you can appropriately increase the sedative and analgesic dose and use muscle relaxants for a short time.

For patients with moderate-to-severe ARDS (PaO2 / FiO2 ≤ 150 mmHg), it is recommended to implement daily prone ventilation for > 12 h as soon as possible.We do not generally recommend strategy of lung expantion due to complications such as barotrauma or pneumothorax.Inhaled nitric oxide were not used in our study.

Maintaining water and electrolyte balance：

In severe and critically ill patients, CRRT was mainly used to maintain water and electrolyte balance and indications for CRRT in COVID-19 patients were as follows:

(1) Patients undergoing maintenance hemodialysis with fever, or suspected, confirmed or clinically diagnosed COVID-19 infection.

(2) Patients combined with AKI, which results in severe electrolyte and acid-base disturbance or volume overload who need blood purification treatment.

(3) Patients with the complications of multiple organ disorder syndrome (MODS), sepsis, severe inflammatory reaction syndrome (SIRS), macrophage activation syndrome (MAS).

(4) Patients with severe disorders of water, electrolyte and acid-base balance caused by other reasons, which are difficult to correct with routine treatment.

(5) Patients with high inflammatory response: serum interlukin-6 (IL-6) > 42pg/ml; and/or interlukin-2 receptor (IL-2R) > 900U/ml; and/or tumor necrosis factor-α (TNF-α) > 48.8pg/ml; and/or high-sensitivity C-reactive protein (hsCRP) >120mg/L.

Restrictive liquid management：

In the case of ensuring the perfusion of vital organs, restrictive fluid management should be implemented to help improve oxygenation and lung injury especially for COVID-19 patients with tracheal intubation.And the protocols based on the following guidelines and references:

1 ARDS: The Berlin Definition

2 A perspective on the Fluids and Catheters Treatment Trial (FACTT): Fluid restriction is superior in acute lung injury and ARDS. N Engl J Med 2006; 354:2564-2574

3 Comparison of Two Fluid-Management Strategies in Acute Lung Injury The National Heart, Lung, and Blood Institute Acute Respiratory Distress Syndrome (ARDS) Clinical Trials Network. N Engl J Med 2006;354(24):2564-75

4 The Importance of Fluid Management in Acute Lung Injury Secondary to Septic Shock. Chest.volume 136, issue 1, July 2009, Pages 102-109
